# An Insect Effector Mimics Its Host Immune Regulator to Undermine Plant Immunity

**DOI:** 10.1002/advs.202409186

**Published:** 2025-01-23

**Authors:** Jianmei Fu, Shuai Li, jing Li, Zhichang Zhao, Jing Li, Xinyang Tan, Shan Yu, Maofeng Jing, Keyan Zhu‐Salzman, Jichao Fang, Rui Ji

**Affiliations:** ^1^ Institute of Plant Protection Jiangsu Academy of Agricultural Sciences Jiangsu Key Laboratory for Food and Safety‐State Key Laboratory Cultivation Base of Ministry of Science and Technology Nanjing 210014 China; ^2^ College of Plant Protection Nanjing Agricultural University Nanjing 210014 China; ^3^ Department of Entomology Texas A&M University, College Station Austin TX 77843 USA

**Keywords:** 14‐3‐3 protein, brown planthopper, rice immunity, effector, mimic

## Abstract

Plants activate defense machinery when infested by herbivorous insects but avoid such costs in the absence of herbivory. However, the key signaling pathway regulators underlying such flexibility and the mechanisms that insects exploit these components to disarm plant defense systems remain elusive. Here, it is reported that immune repressor 14‐3‐3e in rice *Oryza sativa* (OsGF14e) regulates immune homeostasis. Infestation with brown planthopper (BPH) *Nilaparvata lugens* decreased *OsGF14e* expression; however, the level of downregulation is limited both by the short duration and the specific feeding location. OsGF14e interacts with Enhanced Disease Resistance 1‐like (OsEDR1l), a Raf‐like MAP kinase kinase kinase (MAPKKK), and repressed jasmonic acid, jasmonic acid‐isoleucine, and H_2_O_2_ accumulation by enhancing OsEDR1l abundance and signaling ability. OsGF14e and OsEDR1l overexpression renders rice susceptible to BPH, whereas their knockout increases plant resistance but compromises rice growth and grain yield. Intriguingly, BPH 14‐3‐3e protein (Nl14) that shares high sequence homology and structural similarity with OsGF14e is identified from BPH saliva and egg‐associated secretions. Mediated through BPH feeding and oviposition, Nl14, similar to OsGF14e, interacts with OsEDR1l and triggers the OsEDR1l signaling, thereby suppressing plant defenses and facilitating BPH infestation. Apparently, structural and functional mimicry makes it possible for this newly discovered BPH effector to exploit rice OsGF14e‐EDR1l immune suppression module. The results reveal a novel mechanism deployed by herbivorous insects, in a manner similar to certain pathogen effectors, to evade host plant defenses by mimicking host immune regulators.

## Graphical abstract

Under normal conditions, high OsGF14e expression recruits OsEDR1l and enhances OsEDR1l‐mediated MAPK cascade to restrict immunity and promote plant growth. Upon insect infestation, the OsGF14e signaling circuit is rewired toward active immune responses that confer resistance to BPH at the expense of growth. Intriguingly, the effector Nl14 from BPH saliva and egg‐associated secretions mimics OsGF14e, and exploits the OsGF14e‐EDR1l immune suppression module to facilitate BPH infestation.

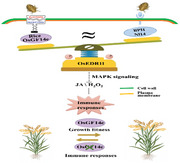

## 1 Introduction

Although plants lack specialized immune cells found in animals, they have developed sophisticated immune‐response systems. When attacked by herbivores, they perceive elicitors derived from the herbivores via specific receptors that cause early signaling events, such as mitogen‐activated protein kinases (MAPKs) activation and reactive oxygen species (ROS) burst.^[^ ^1^ ^]^ These early signaling events lead to the production of defense‐related phytohormones, such as jasmonic acid (JA) and salicylic acid (SA), which are well known to regulate the production of defensive compounds and thus confer resistance to herbivores.^[^ ^1^, ^2^, ^3^ ^]^ Conversely, herbivores evolve various counteracting strategies. Secretion of effectors into host plant cells is an example of such a strategy employed by insects to evade plant defenses.^[^ ^1^, ^4^ ^]^

Constitutive immune responses are costly and often restrict plant growth and crop yield.^[^ ^5^, ^6^, ^7^ ^]^ In contrast, rapid and precise defense responses to herbivore infestation allow for the most efficient use of resources and minimize plant growth penalties.^[^ ^8^ ^]^ An energy‐efficient immune system is particularly advantageous for field crops that are frequently challenged by diverse pathogens and herbivores.^[^ ^9^, ^10^ ^]^ For instance, the immune repressor RESISTANCE OF RICE TO DISEASES1 (ROD1) encodes a Ca^2+^ sensor to suppress rice immunity by promoting ROS scavenging, and its protein stability is fine‐tuned by ubiquitination to avoid excessive immune responses and help equilibrate rice immunity and growth.^[^ ^7^ ^]^

As ubiquitous eukaryotic signaling components, the 14‐3‐3 proteins are involved in multiple physiological processes, including primary metabolism, cell division, apoptosis, and biotic and abiotic stresses.^[^ ^11^ ^]^ These proteins can regulate enzyme activity, enhance stability, or alter the subcellular localization of their target proteins, typically by binding to phosphorylated “client” proteins.^[^ ^12^ ^]^ Increasing evidence indicates that 14‐3‐3 proteins play a crucial role in plant‐microbe interactions.^[^ ^13^ ^]^ The tomato (*Solanum lycopersicum*, *S. lycopersicum*) 14‐3‐3 protein TFT7 interacts with MAPK kinase kinase α (MAPKKKα) and enhances the protein abundance and signaling ability of MAPKKKα, thus causing a hypersensitive response and enhanced disease resistance. Moreover, three amino acid residues (R58‐R130‐Y131) in TFT7 likely form a contact surface for binding to MAPKKKα. ^[^ ^14^ ^]^ Direct interaction of the *Arabidopsis* 14‐3‐3 proteins GRF6 and GRF8 with MAPKKK5 enables its activation to be controlled by upstream RLCKs, resulting in enhanced antibacterial and antifungal immunity.^[^ ^15^ ^]^ In addition, several 14‐3‐3 proteins have been identified as direct targets of effectors from plant pathogens and herbivorous insects in regulating plant immunity,^[^ ^16^, ^17^, ^18^, ^19^ ^]^ as exemplified by the non‐TAL effector protein Xanthomonas outer protein Q (XopQ) and the aphid effector protein Me10.^[^ ^18^, ^19^ ^]^ Nevertheless, information regarding 14‐3‐3 protein‐regulated plant defense signaling against insects is limited.

As a common target of 14‐3‐3, MAPKKK initiates the activation of MAPK cascade signaling through sequential phosphorylation in a three‐tiered system: MAPKKK phosphorylates MAPKK, which further phosphorylates MAPK, and then the activated MAPK phosphorylates multifarious proteins to activate cellular responses.^[^ ^20^ ^]^ The rice genome encodes 75 MAPKKK‐like kinases, 43 of which are Raf‐like kinases.^[^ ^21^ ^]^ Raf‐like kinases are involved in diverse developmental and physiological processes, such as biotic and abiotic responses, hormone signal transduction, and embryonic development.^[^ ^22^, ^23^ ^]^ Accumulating data, mostly from studies focusing on plant‐microbe interactions, have demonstrated that Raf‐like MAPKKKs play central roles in plant immunity. ENHANCED DISEASE RESISTANCE 1 (EDR1), a Raf‐like MAPKKK, suppresses SA‐ and JA‐associated defense signaling, rendering rice susceptible to *X. oryzae* pv. *Oryzae*.^[^ ^23^ ^]^ Moreover, the downstream kinase MPK3 negatively mediates rice resistance to the blast fungus *Magnaporthe oryzae* by suppressing JA signaling.^[^ ^24^ ^]^ Although a phloem‐derived EDR1‐like protein in tobacco (*Nicotiana tabacum*) was found to trigger phloem‐localized ROS burst to inhibit aphid feeding,^[^ ^25^ ^]^ the mechanisms underlying the ability of Raf‐like MAPKKKs to regulate plant defenses against insects remain largely unknown.

Intriguingly, saliva proteome analyses have revealed that 14‐3‐3e, a conserved salivary protein, is found in multiple piercing‐sucking herbivorous arthropods, such as whiteflies, spider mites, triatomines, and rice planthoppers.^[^ ^26^, ^27^, ^28^, ^29^ ^]^ Recently, we have also identified 14‐3‐3e in the proteome of egg‐associated secretions from brown planthopper (BPH) *Nilaparvata lugens* using liquid chromatography‐tandem mass spectrometry analysis,^[^ ^30^ ^]^ implying that BPH 14‐3‐3e (Nl14) can come into contact with rice tissues during feeding and oviposition. However, the function of the herbivore‐derived effector candidate 14‐3‐3e remains unclear. Thus, we conducted a study to track the relationship between the rice immune regulator 14‐3‐3e (OsGF14e) and the BPH salivary and egg‐associated effector 14‐3‐3e (Nl14), and illustrated how BPH Nl14 exploits its host immune suppression system by mimicking OsGF14e. Our study reveals a previously unrecognized mechanism whereby plant and herbivore factors suppress plant immune responses through the same cascade.

## 2 Results

### 2.1 BPH Infestation Dynamically Downregulates Rice OsGF14e Expression

**Figure 1:**
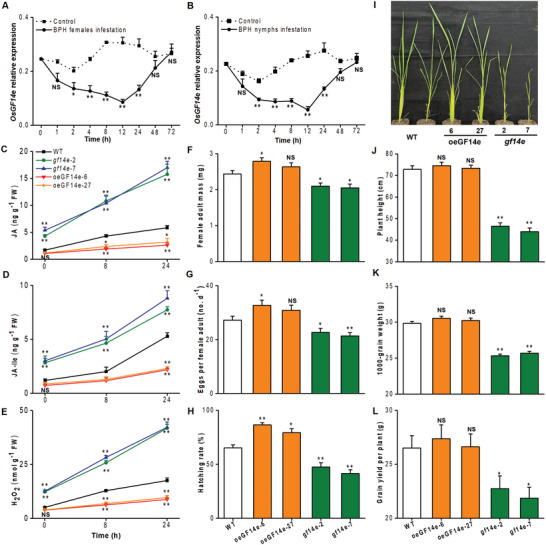
OsGF14e affects rice growth and resistance to BPH. A,B) Mean transcript levels ± SEM (*n* = 3) of *OsGF14e* in the feeding sites of rice plants that were infested by either BPH gravid female adults (A) or 5th instar nymphs (B). Controls correspond to non‐manipulated plants. Asterisks indicate significant differences between treatments and controls (*, *p* < 0.05; **, *p* < 0.01; NS, not significant; Student's *t*‐test). C–E) Mean levels ± SEM (*n* = 6) of JA, JA‐Ile, and H_2_O_2_ in *gf14e* mutants, oeGF14e, and WT plants that were individually infested by BPH gravid female adults. F) Mean mass ± SEM (*n* = 10) of a newly emerged BPH female adult feeding on *gf14e* mutants, oeGF14e, and WT plants. G) Mean number ± SEM (*n* = 9–15) of eggs laid by a BPH female adult on *gf14e* mutants, oeGF14e, and WT plants. H) Mean hatching rate (*n* = 9–15) of BPH eggs on *gf14e* mutants, oeGF14e, and WT plants. I) Growth phenotypes of *gf14e* mutants, oeGF14e, and WT plants. J‐L) Mean plant height ± SEM (*n* = 10, J), 1000‐grain weight ± SEM (*n* = 15, K) and grain weight per plant ± SEM (*n* = 15, L) of *gf14e* mutants, oeGF14e, and WT plants. Asterisks indicate significant differences between WT and transgenic plants in Figure 1C–H,J–L (*, *p* < 0.05; **, *p* < 0.01; NS, not significant; Student's *t*‐test). Error bars represent standard errors.

**Figure 2:**
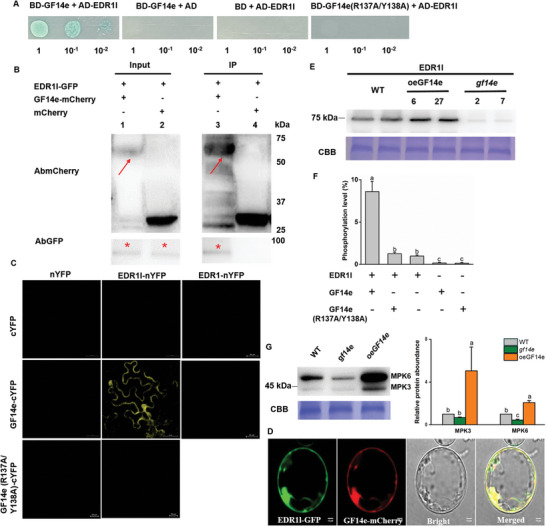
Interactions between OsGF14e and OsEDR1l. A) Yeast‐two hybrid (Y2H) assay of yeast strain co‐transformed with the indicated plasmids and spotted on quadruple dropout media with X‐a‐gal. Ala substitutions were made at R137 and Y138 positions of OsGF14e to generate OsGF14e (R137A/Y138A). B) Co‐immunoprecipitation (Co‐IP) analysis. OsEDR1l‐GFP and OsGF14e‐mCherry fusion protein were transiently co‐expressed in *Nicotiana benthamiana*. Protein extracts (input) were immunoprecipitated by mCherry‐trap (mRFP‐Trap) beads (IP) and resolved by SDS‐PAGE. Immunoblots were developed with mCherry antibody (AbmCherry) to detect OsGF14e‐mCherry protein (arrowheads) and with green fluorescent protein (GFP) antibody (AbGFP) to detect OsEDR1l‐GFP (asterisks). The Co‐IP assays were repeated three times. C) Bimolecular fluorescence complementation (BiFC) analysis to determine the interaction in the leaf cells of *N. benthamiana*. Fluorescence indicates reconstitution of an intact fluorescent protein from the complementary OsGF14e‐cYFP and OsEDR1l‐nYFP (yellow fluorescent protein). A previous studied OsEDR1 (ref. [23], LOC_Os03g06410) served as a negative control. D) Co‐localization of OsGF14e and OsEDR1l to the cytoplasm and nucleus. OsEDR1l‐GFP and OsGF14e‐mCherry fusion protein were co‐expressed in rice protoplasts by PEG‐mediated transformation. Confocal laser‐scanning microscopy was used to investigate their distribution 24 h after transformation. OsEDR1l (green) and OsGF14e (red) were mainly co‐localized in the cytoplasm and nucleus (yellow). E‐G) OsEDR1l expression in *gf14e* mutants, oeGF14e and WT plants (E), OsEDR1l phosphorylation level in vitro (F), OsMPK3/6 phosphorylation levels in *gf14e* mutant, oeGF14e and WT plants (G). In vitro analysis of OsEDR1l phosphorylation level was performed using recombinant OsEDR1l and oeGF14 proteins, and recombinant OsGF14e (R137A/Y138A) protein was used as the negative control (F). Phospho‐p44/42 MAPK (Thr202/Tyr204) antibodies were used for phosphorylation test, and the Western blot band density of phosphorylated OsMPK3/6 protein in *gf14e* mutant and oeGF14e plants (relative to WT) was quantified from three biological replicates using ImageJ software (G). The rice plants were infested by BPH for 12 h. CBB staining was shown as the protein loading control, OsEDR1l phosphorylation (F), Each assay was repeated three times. Different letters indicate a significant difference among treatments (*p* < 0.05; one‐way ANOVA followed by Duncan's multiple range test). Error bars represent standard errors.

Given that 14‐3‐3 proteins play a crucial role in plant‐pathogen interactions, we measured the expression patterns of all rice *14‐3‐3* gene members in response to BPH infestation. Among the eight members, only *OsGF14e* and *OsGF14* *h* displayed consistent and significant alterations in expression within 48 h of BPH infestation (**Figure** **1**A; Figure S1, Supporting Information). OsGF14e is well known to negatively affect ROS accumulation and promote broad‐spectrum disease susceptibility in rice.^[^ ^31^ ^]^ Therefore, we investigated the potential role of OsGF14e in rice response to BPH infestation. Both BPH female adults and 5^th^‐instar nymphs caused a rapid and dramatic decrease in *OsGF14e* transcripts, with the lowest levels reached at 12 h of infestation (Figure 1A,B). The suppressive effect then weakened and ultimately returned to the control level at 48 to 72 h (**Figure** **2**A). Moreover, changes in gene expression were mainly limited to BPH feeding sites (Figure S2, Supporting Information). Mechanical wounding alone decreased the transcription levels of *OsGF14e* at 12 and 24 h after treatment (Figure S2, Supporting Information). These results suggest that OsGF14e is an early as well as local and transitory regulator of rice responses to BPH.

### 2.2 OsGF14e Affects Rice Immunity and Growth

To understand how OsGF14e influences defense signaling in rice, two independent OsGF14e knockout (*gf14e*) mutants and two transgenic lines overexpressing *OsGF14e* (oeGF14e) in the *Nipponbare* genetic background (wild‐type, WT) were generated. The *gf14e* mutants had either a 1‐bp insertion or a 1‐bp deletion at different target sites, resulting in frame shifts and producing a premature termination in the protein sequence (Figure S3A,B, Supporting Information). Moreover, they still contain T‐DNA through detecting a hygromycin resistance (*hptII*) gene. The oeGF14e lines were confirmed by measuring *OsGF14e* transcript levels (Figure S3C, Supporting Information). JA‐ and hydrogen peroxide (H_2_O_2_) ‐mediated signaling pathways play a central role in enhancing rice resistance to BPH.^[^ ^4^, ^32^ ^]^ Thus, we compared the anti‐herbivore signaling levels in *OsGF14e* transgenic lines with those in WT plants. The levels of JA, JA‐Ile, and H_2_O_2_ were significantly higher in the *gf14e* mutants than the WT plants with or without BPH attack. Conversely, they were only significantly lower in the BPH‐infested oeGF14e lines compared with the BPH‐infested WT plants (Figure 1C–E). Whereas, the SA levels did not significantly change in the *OsGF14e* transgenic lines compared with WT plants (Figure S3D, Supporting Information). Thus, OsGF14e appears to repress BPH‐induced JA, JA‐Ile, and H_2_O_2_ production.

An evaluation of resistance to BPH in different OsGF14e transgenic rice lines showed that female adults gained less mass when fed the two *gf14e* mutant lines and more mass when fed the oeGF14e line compared with those fed the WT plants (Figure 1F). The same trend was observed for the reproductive parameter, with fewer eggs laid on the two mutant lines and more eggs laid on the oeGF14e line (Figure 1G). Moreover, egg hatching rates were lower for BPH on the *gf14e* mutants and higher for BPH on the oeGF14e lines compared with those on the WT controls (Figure 1H). Consistently, oeGF14e lines were more severely damaged by BPH than the WT plants, whereas the *gf14e* mutants were less damaged (Figure S4A, Supporting Information). Taken together, OsGF14e represses rice defense responses, likely by negatively regulating JA, JA‐Ile, and H_2_O_2_ accumulation.

We examined the role of OsGF14e in plant growth and grain yield. Knocking out *OsGF14e* resulted in dwarf plants (Figure 1I,J) that exhibited poor agronomic traits, including significantly lower grain yield (Figure 1K,L). However, the oeGF14e lines exhibited growth and agronomic traits comparable to those of the WT plants (Figure 1I–L). Thus, when coping with BPH infestation, the presence of OsGF14e likely prevents substantial unnecessary fitness costs in rice.

### 2.3 OsGF14e Interacts with OsEDR1l and Enhances OsEDR1l Abundance

To investigate the molecular mechanism by which OsGF14e regulates induced defense in rice, we attempted to identify OsGF14e‐targeted host factors using yeast two‐hybrid (Y2H) experiments. Using OsGF14e as the bait, we screened a rice cDNA library. From approximately 3.7 million primary yeast transformants, we obtained 31 distinct positive clones on quadruple nutrient‐deficient medium, and they represented 19 different target genes (Table S1, Supporting Information), including the serine/threonine‐protein kinase EDR1‐like (OsEDR1l). A previous study demonstrates that OsEDR1l is a member of the Raf‐like MAPKKK family, characterized by a conserved protein kinase domain and a signature motif (GTPEWMAPEL).^[^ ^21^ ^]^ We selected this MAPKKK for further analysis because of its putative function as an immunity‐related protein. A blast search of the NCBI database identified two predicted isoforms of *OsEDR1l* from japonica rice: X1 (XM_015770802.2) and X2 (XM_015770804.2). Isoform X1 may encode a protein with 790 amino acids (88.20 kDa), while X2 may encode a protein with 662 amino acids (74.48 kDa). We used their conserved peptide CGPDEQEGSRRQVSN to generate a polyclonal antibody. However, western blot analysis revealed one OsEDR1l‐related band (≈75 kDa) in japonica rice varieties *Nipponbare* and Zhonghua 11 (ZH11), indicating that isoform X2 is predominant (Figure S5A, Supporting Information). Consequently, we selected OsEDR1l isoform X2 for further experiments. The amino acid sequences of both OsGF14e and OsEDR1l are predicted to have nuclear localization signals (Figures S3B and S14B, Supporting Information). Subcellular localization of OsEDR1l‐GFP (GFP, green fluorescent protein) via transient expression in rice protoplasts suggested that it was distributed in both the cytoplasm and nucleus (Figure S6, Supporting Information).

Yeast transformants co‐expressing the DNA‐binding domain (BD)‐OsGF14e and activating domain (AD)‐OsEDR1l were able to grow on quadruple dropout medium, whereas transformants bearing the control or some other OsEDR1 members constructs were not (Figure 2A; Figures S7 and S8A, Supporting Information). Furthermore, the OsEDR1l‐GFP (GFP, green fluorescent protein) fusion protein was co‐precipitated with OsGF14e‐mCherry (mCherry, red fluorescent protein) when transiently co‐expressing OsEDR1l‐GFP and OsGF14e‐mCherry in *N. benthamiana*, indicating *in planta* interactions between OsGF14e and OsEDR1l (Figure 2B). Their association was confirmed by bimolecular fluorescence complementation (BiFC) assay, as evidenced by the observation of yellow fluorescence when transiently co‐expressing OsGF14e‐cYFP (cYFP, C‐terminal of yellow fluorescent protein) and OsEDR1l‐nYFP (nYFP, N‐terminal of yellow fluorescent protein) in *N. benthamiana* (Figure 2C). Finally, OsGF14e‐mcherry and OsEDR1l‐GFP colocalized in both the cytoplasm and nucleus (Figure 2D).

OsEDR1l contains three domains, EDR1, FRG2, and Ser/Thr kinase. Y2H assay showed that OsGF14e interacted with Ser/Thr kinase within OsEDR1l (Figure S9, Supporting Information). Moreover, a previous study demonstrated that R131 and Y132 in the phosphopeptide‐binding motif of the tomato 14‐3‐3 protein TFT7 are essential for its binding to MAPKKKɑ.^[^ ^14^ ^]^ Using a similar approach, alanine (Ala) substitutions were introduced at the corresponding positions, R137 and Y138, of OsGF14e to generate the OsGF14e (R137A/Y138A) variant. Through Y2H and BiFC assays, we observed that OsGF14e (R137A/Y138A) failed to interact with OsEDR1l (Figure 2A,C and Figure S10, Supporting Information), indicating that the R137 and Y138 residues are critical for its interaction with OsEDR1l.

Phylogenetic analysis of all MAPKKK in rice placed OsEDR1l on a distinct branch (Figure S5B, Supporting Information). To determine the specificity of OsEDR1l interaction with OsGF14e, we assessed whether other OsEDR1 homologs in rice could interact with OsGF14e. Both Y2H and BiFC experiments showed that the selected eight OsEDR1 members could not interact with GF14e (Figure 2C; Figure S8, Supporting Information).

Furthermore, we investigated whether OsGF14e influences the transcript levels and protein abundance of OsEDR1l. Since a significant difference was not observed in *OsEDR1l* gene expression among the WT, *gf14e* mutant, and oeGF14e lines, OsGF14e might not affect *OsEDR1l* expression at the transcriptional level (Figure S11, Supporting Information). In contrast, the protein abundance of OsEDR1l increased in rice overexpressing OsGF14e but was reduced in *gf14e* mutants (Figure 2E). Moreover, recombinant OsEDR1l, OsGF14, and OsGF14e (R137A/Y138A) proteins were obtained by expressing them in *Escherichia coli* (Figure S12, Supporting Information). In vitro analysis of OsEDR1l phosphorylation levels revealed that purified OsGF14e enhanced the phosphorylation of purified OsEDR1l, but OsGF14e (R137A/Y138A) did not (Figure 2F). Reports have shown that 14‐3‐3 proteins stabilize their target proteins by preventing protease‐mediated degradation. ^[^ ^12^, ^14^ ^]^ In our experiments, co‐expression of OsEDR1l‐GFP and OsGF14e‐mCherry i*n N. benthamiana* leaves resulted in increased accumulation of OsEDR1l compared to co‐expression of OsEDR1l‐GFP and mCherry, whereas treatment with the proteasome inhibitor MG132 eliminated the observed difference in OsEDR1l accumulation (Figure S13, Supporting Information). Thus, OsGF14e promotes the expression of OsEDR1l, likely by post‐translationally stabilizing the protein and inhibiting its degradation.

To further determine whether this could affect the activity of downstream MAPKs, we measured the activity of MPK3/MPK6 in WT and transgenic lines. Phosphorylation of MPK3/MPK6 was higher in the oeGF14e plants than in the WT plants, while that of MPK6 was lower in the *gf14e* mutant (Figure 2G). Therefore, OsGF14e enhanced the protein accumulation and phosphorylation level of OsEDR1l, and promoted the downstream MAPKs signaling.

### 2.4 OsEDR1l Represses Rice Defense Against BPH

**Figure 3:**
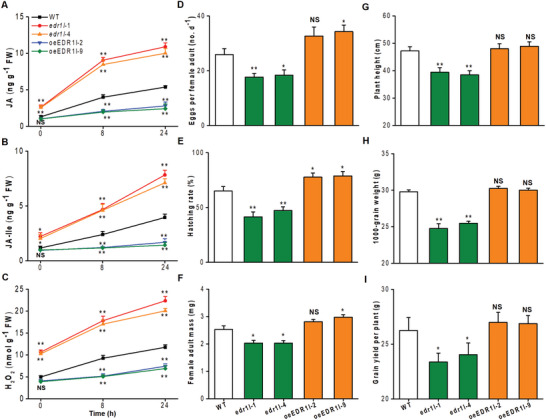
OsEDR1l modulates insect resistance and plant growth in rice. A–C) Mean levels ± SEM (*n* = 6) of JA, JA‐Ile, and H_2_O_2_ in *edr1l* mutants, oeEDR1l, and WT plants that were individually infested by BPH. D) Mean number ± SEM (*n* = 9–15) of eggs laid by a BPH female adult on *edr1l* mutants, oeEDR1l and WT plants. E) Mean hatching rate ± SEM (*n* = 9–15) of BPH eggs on *edr1l* mutants, oeEDR1l and WT plants. F) Mean weight ± SEM (*n* = 10) of a newly emerged BPH female adult on *edr1l* mutants, oeEDR1l and WT plants. G‐I) Mean plant height ± SEM (*n* = 10, G), 1000‐grain weight (*n* = 15, H), and grain weight per plant (*n* = 15, I) of *edr1l* mutants, oeEDR1l, and WT plants. Asterisks indicate significant differences between WT and transgenic rice plants (*, *p* < 0.05; **, *p* < 0.01; NS, not significant; Student's *t*‐test) with error bars representing standard errors.

A study has reported that a OsEDR1 homolog negatively regulates rice bacterial resistance by affecting the production of defense‐related phytohormones.^[^ ^23^ ^]^ However, amino acid sequence alignment of the reported OsEDR1 and our OsEDR1l shared low sequence homology (23.8% identity). To elucidate the role of novel OsEDR1l in insect resistance regulation, two independent OsEDR1l knockout mutants (*edr1l* lines) and two overexpression lines (oeEDR1l lines) were generated in the *Nipponbare* genetic background (wild‐type, WT). The *edr1l* mutants had either a 1‐bp insertion or a 2‐bp deletion at the two target sites, resulting in frame shifts and producing a premature termination in the protein sequence (Figure S14A and B, Supporting Information). Moreover, they still contain T‐DNA through detecting the *hptII* gene. The oeEDR1l lines were also confirmed by the quantification of *OsEDR1l* transcript levels (Figure S14C, Supporting Information). To understand how OsEDR1l influences anti‐insect signaling in rice, we examined JA, JA‐Ile, and H_2_O_2_ levels in these lines. They were higher in the *edr1l* mutants with or without BPH infestation and lower in the BPH‐infested oeEDR1l lines than in the WT plants (**Figure** **3**A‐C). These experiments demonstrate that OsEDR1l negatively regulates JA, JA‐Ile, and H_2_O_2_ production, which is similar to the effects of OsGF14e.

An evaluation of resistance to BPH in different rice lines showed that BPH female adults laid fewer eggs on the two mutant lines and more eggs laid on the oeEDR1l line than on the WT plants (Figure 3D). The same trend was observed for egg hatching rate, female adult mass, and plant damage phenotype (Figure 3E,F and Figure S4B, Supporting Information). These results strongly indicate that OsEDR1l is likely a repressor of BPH resistance. Also similar to OsGF14e loss‐of‐function lines, the OsEDR1l knockout resulted in dwarfed plants (Figs. 3G; Figure S14D, Supporting Information) that exhibited poor agronomic traits (Figure 3H,I).

### 2.5 BPH Effector Nl14 Mimics OsGF14e and Exploits OsGF14e‐EDR1l Immune Suppression Module in Rice

**Figure 4:**
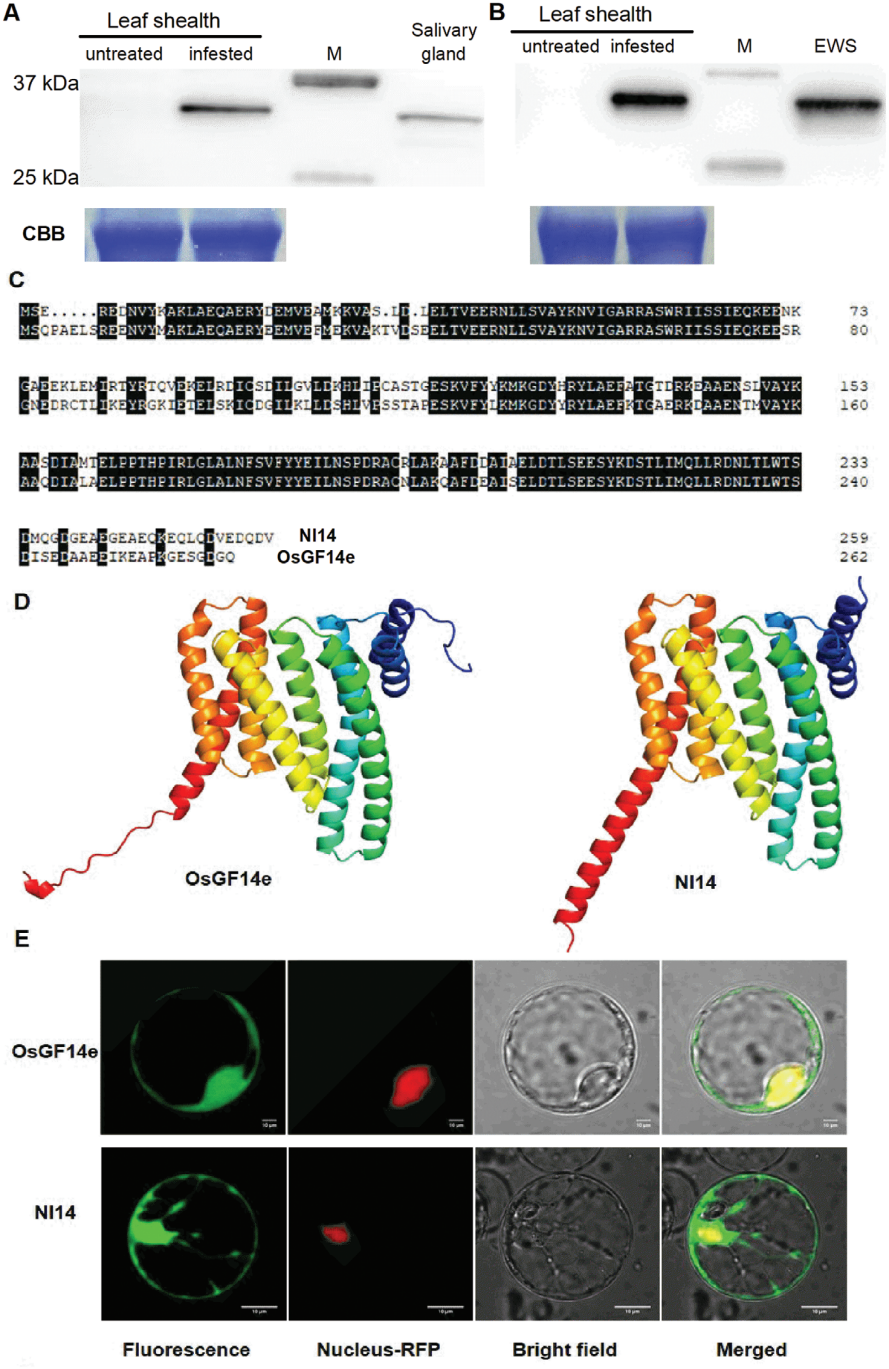
High sequence homology and structural and cellular localization similarity between OsGF14e and BPH Nl14. A‐B) BPH Nl14 protein enters rice tissues during BPH feeding (A) and eggs deposition (B). A) Western blotting analysis showing that Nl14 is present in leaf sheaths that were infested for 24 h by 50 newly emerged BPH female adults (feeding only, infested) and in salivary gland, but not in non‐infested leaf sheaths. B) Western blotting analysis showing that Nl14 is present in leaf sheaths that were infested by 20 BPH gravid females without stylets (oviposition only, infested) for 24 h but BPH eggs were removed, and in the intact BPH eggs wash solution (EWS) respectively, but not in non‐infested leaf sheaths. The stylets of gravid BPH females were removed under a microscope to eliminate the effect of feeding. M, molecular weight markers (kDa). C) Full‐length amino acid sequences alignment of Nl14 and OsGF14e. Identical amino acids were shaded black. The positions of the amino acid substitutions are underlined in red. D) Three‐dimensional structure of OsGF14e and Nl14. E) Cellular localization of OsGF14e or Nl14 in rice protoplasts. GFP fusion proteins of OsGF14e and Nl14 were expressed in rice protoplasts by polyethylene glycol‐mediated transformation. Confocal laser‐scanning microscopy was used to investigate their distribution 16 h post‐transformation.

Two 14‐3‐3 proteins, 14‐3‐3e (Nl14) and 14‐3‐3z, were detected in the saliva of planthoppers.^[^ ^27^ ^]^ When expressed individually in *N. benthamiana* leaves, Nl14 significantly alleviated the fungal elicitor chitosan‐induced cell death, whereas 14‐3‐3z did not (Figure S15, Supporting Information). Based on these results, we selected Nl14 for further investigation of its effector function. Analysis of gene expression in different tissues and insect developmental stages revealed that *Nl14*, while was expressed at all stages, was most abundant in the salivary glands, followed by the oviducts (Figure S16, Supporting Information). To determine whether Nl14 is secreted into rice tissues during BPH feeding and oviposition, we performed western blot analysis using an Nl14‐specific peptide antibody. A band of ≈30 kDa was detected in the total proteins isolated from rice leaf sheaths fed by newly emerged BPH female adults for 24 h (feeding only) but not from non‐infested plants (**Figure** **4**A). Thus, Nl14 is secreted into rice tissues via feeding. Additionally, to eliminate potential interference from Nl14 protein in the saliva, gravid BPH females with their stylets removed were allowed to lay eggs on rice leaf sheaths. After 24 h, intact BPH eggs were carefully collected. An Nl14 band was subsequently detected in the total proteins isolated from the infested leaf sheaths, although it was not detected in the total protein from non‐infested plants (Figure 4B). This band was also detected in the intact egg wash solution (EWS; Figure 4B), suggesting that Nl14 was present on the surface of BPH eggs and/or in oviposition fluids and thus could come into contact with damaged rice tissues during oviposition.

**Figure 5:**
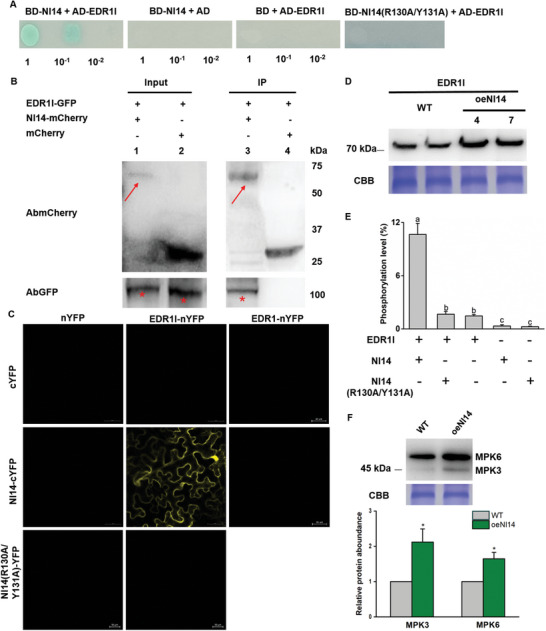
Interactions between Nl14 and OsEDR1l. A) Y2H assay of yeast strain co‐transformed with the indicated plasmids and spotted on quadruple dropout medium with X‐a‐gal. Ala substitutions were made at R130 and Y131 positions of Nl14 to generate Nl14 (R130A/Y131A). B) Co‐IP analysis. OsEDR1l‐GFP and Nl14‐mCherry fusion protein were transiently co‐expressed in *Nicotiana benthamiana*. Protein extracts (input) were immunoprecipitated by mCherry‐trap (mRFP‐Trap) beads (IP) and resolved by SDS‐PAGE. Immunoblots were developed with mCherry antibody (AbmCherry) to detect Nl14‐mCherry protein (arrowheads) and with green fluorescent protein (GFP) antibody (AbGFP) to detect OsEDR1l‐GFP (asterisks). C) Bimolecular fluorescence complementation (BiFC) analysis to determine the interaction in vivo. Fluorescence indicates reconstitution of an intact fluorescent protein from the complementary Nl14 ‐cYFP and OsEDR1l‐nYFP (yellow fluorescent protein). A previous studied OsEDR1 (ref. [23], LOC_Os03g06410) served as a negative control. D‐F) OsEDR1l expression in oeNl14 and WT plants (D), OsEDR1l phosphorylation level in vitro (E), and OsMPK3/6 phosphorylation levels in oeNl14 and WT plants (F). In vitro analysis of OsEDR1l phosphorylation level was performed using recombinant OsEDR1l and Nl14 proteins, and the recombinant Nl14 (R130A/Y131A) protein was used as the negative control (E). Phospho‐p44/42 MAPK (Thr202/Tyr204) antibodies were used for OsMPK3/6 phosphorylation test, and the Western blot band density of phosphorylated OsMPK3/6 protein in oeNl14 plants (relative to WT) was quantified from three biological replicates using ImageJ software (F). The rice plants were infested by BPH for 12h. CBB staining was shown as the protein loading control. Asterisks indicate significant differences between WT and oeNl14 plants (*, *p* < 0.05; Student's *t*‐test) with error bars representing standard errors.

Given that protein function is largely determined by the protein sequence, three‐dimensional (3D) conformation, and cellular localization, the similarities between rice OsGF14e and BPH candidate effector Nl14 were investigated. Their amino acid sequence alignment showed 76% identity (Figure 4C). Structure homology modeling indicated that both molecules consisted of nine ɑ‐helices and shared a similar 3D structure (Figure 4D). When transiently expressed individually in rice protoplasts, both proteins were detected in the plant cytoplasm and nucleus (Figure 4E). To elucidate whether Nl14 also interacts with OsEDR1l, two independent Nl14 overexpressing lines (oeNl14 lines) in the *Nipponbare* genetic background were generated, with similar growth phenotypes to WT plant (Figure S17, Supporting Information). The oeNl14 lines were confirmed by the quantification of Nl14 transcript levels, and *Nl14* overexpression did not affect *OsGF14e* expression in rice plant (Figure S18, Supporting Information). The protein‐protein interaction assays showed that Nl14 could interact with OsEDR1l through Y2H, Co‐Immunoprecipitation, and BiFC experiments (**Figure** **5**A‐C), but could not interact with some other OsEDR1 members in rice (Figure 5C; Figure S8, Supporting Information). Moreover, Ala substitutions were also introduced at the R131 and Y132 positions of Nl14, generating the Nl14 (R131A/Y132A) variant. Using Y2H and BiFC assays, we found that Nl14 (R131A/Y132A) failed to interact with OsEDR1l (Figure 5A,B and Figure S10, Supporting Information), indicating that the R131 and Y132 residues are critical for its interaction with OsEDR1l.

Further results showed that, similar to OsGF14e, Nl14 could enhance the abundance of OsEDR1l protein as well as phosphorylation levels of purified OsEDR1l and MPK3/6 (Figure 5D–F). Notably, Nl14 (R131A/Y132A) also could not enhance the phosphorylation of purified OsEDR1l (Figure 5E). Most likely, the high similarity in the primary sequence, 3D conformation, cellular localization, and the target protein in rice rendered it possible for Nl14 to perform OsGF14 functions once secreted into plant cells.

**Figure 6:**
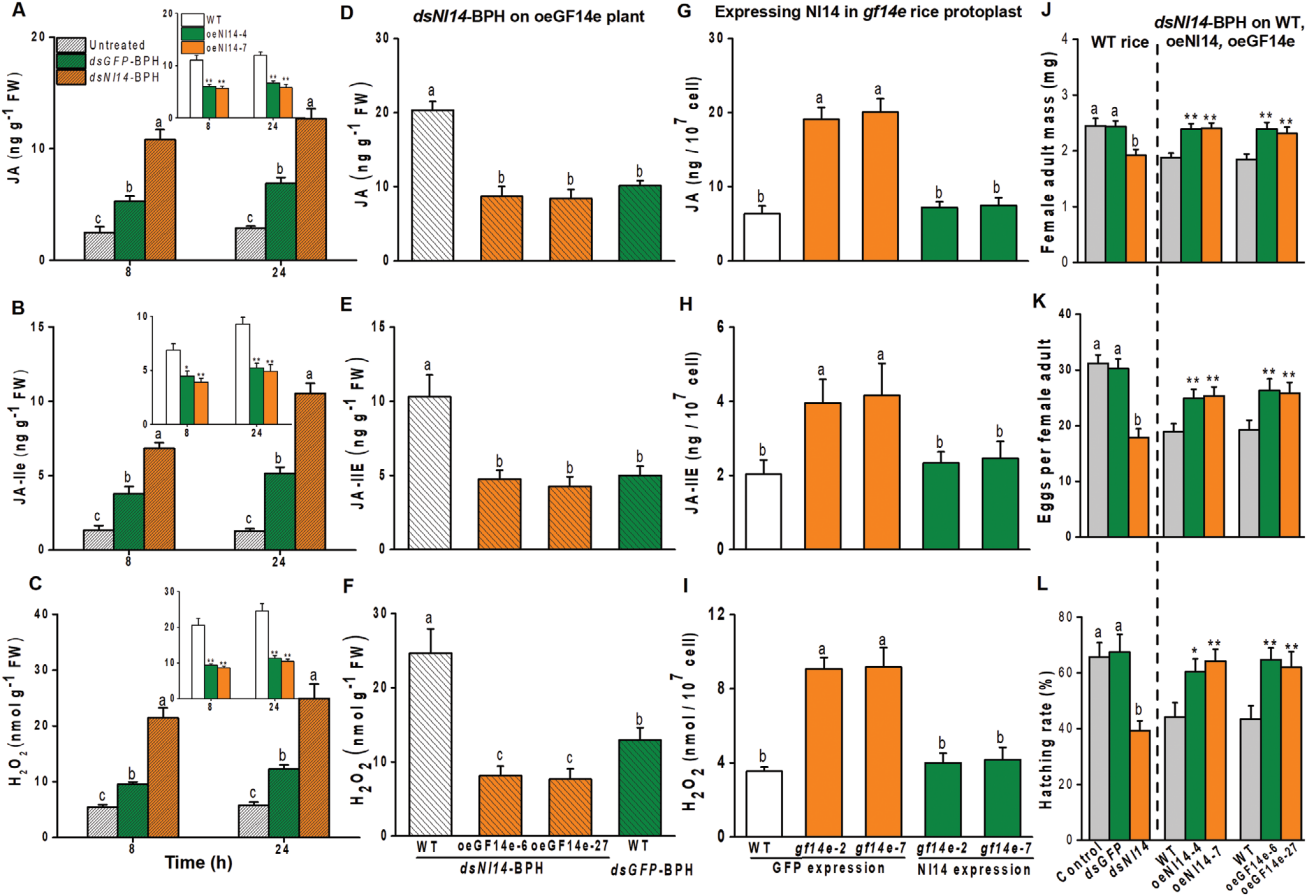
Functional similarity between the BPH effector Nl14 and rice immune repressor OsGF14e.A‐C) Mean levels ± SEM (*n* = 6) of JA, JA‐Ile, and H_2_O_2_ in rice plants after they were untreated or infested individually by BPH gravid female adults, which had been injected 2 d earlier with either Nl14 dsRNA (*dsNl14*‐BPH) or GFP dsRNA (*dsGFP*‐BPH). Inserts: Mean levels ± SEM (*n* = 6) of JA, JA‐Ile, and H_2_O_2_ in oeNl14 lines and WT plants that were individually infested by *dsNl14*‐BPH. D‐F) Mean levels ± SEM (*n* = 6) of JA, JA‐Ile, and H_2_O_2_ in oeGF14e lines and WT plants that were individually infested by *dsNl14*‐BPH or *dsGFP*‐BPH. G‐I) Mean levels ± SEM (*n* = 6) of JA, JA‐Ile, and H_2_O_2_ in the protoplasts of *gf14e* mutants and WT rice plants. Nl14‐GFP was transiently expressed in rice protoplasts via polyethylene glycol‐mediated transfection transformation. Expressing GFP served as a vector control. J) Left lane: Mean levels ± SEM (*n* = 10) of a newly emerged female adult of untreated BPH or *dsNl14*‐BPH or *dsGFP*‐BPH. Right two lanes: Mean levels ± SEM (*n* = 10) of a newly emerged *dsNl14*‐BPH female adult on the oeNl14, oeGF14e or WT plants. K) Left lane: Mean levels ± SEM (*n* = 9–15) of eggs laid by an untreated BPH or *dsNl14*‐BPH or *dsGFP*‐BPH on WT plants. Right two lanes: Mean levels ± SEM (*n* = 9–15) of eggs laid by a *dsNl14*‐BPH on the oeNl14, oeGF14e, or WT plants. L) Left lane: Mean hatching rates ± SEM (*n* = 9–15) of eggs laid by untreated BPH (control) or *dsNl14*‐BPH or *dsGFP*‐BPH on WT plants. Right two lanes: Mean hatching rate ± SEM (*n* = 9–15) of eggs laid by *dsNl14*‐BPH on oeNl14, oeGF14e or WT plants. Asterisks indicate significant differences between transgenic and WT plants (*, *p* < 0.05; **, *p* < 0.01; Student's *t*‐test). Different letters indicate a significant difference among treatments (*p*< 0.05, one‐way ANOVA followed by Duncan's multiple range test). Error bars represent standard errors.

To further illustrate the effector function of Nl14, we measured the concentrations of JA, JA‐Ile, and H_2_O_2_ in rice plants untreated or infested with *GFP* dsRNA‐injected BPH (*dsGFP*‐BPH) or *Nl14* dsRNA‐injected BPH (*dsNl14*‐BPH) to determine whether secreted Nl14 could influence biosynthesis of these compounds. Injection with *Nl14* dsRNA decreased *Nl14* transcript levels in BPH by 76–88% at 2–8 days post‐injection (Figure S19, Supporting Information). The levels of JA, JA‐Ile, and H_2_O_2_ were significantly higher in *dsNl14*‐BPH‐infested rice plants than in *dsGFP*‐BPH‐infested plants (**Figure** **6**A–C). After rice plants were infested with *dsNl14*‐BPH, the JA, JA‐Ile, and H_2_O_2_ levels were significantly lower in the oeNl14 plants compared with the WT plants (inserts in Figure 6A–C). Thus, the BPH effector Nl14 suppresses BPH‐induced JA, JA‐Ile, and H_2_O_2_ accumulation in rice.

We further investigated the functional similarity between Nl14 and OsGF14e. After rice plants were infested with *dsNl14*‐BPH, levels of JA, JA‐Ile, and H_2_O_2_ were significantly lower in oeGF14e plants compared to WT plants, reaching a level comparable to that in WT plants infested with *dsGFP*‐BPH (Figure 6D‐F). These results suggest that overexpression of OsGF14e in rice can suppress *dsNl14*‐BPH‐induced defense signaling. In contrast, when Nl14 was expressed in rice protoplasts of *gf14e* mutants, the accumulation of JA, JA‐Ile, and H_2_O_2_ was significantly reduced, reaching levels similar to those in WT rice protoplasts expressing GFP (Figure 6G–I). This indicates that Nl14 can suppress defense signaling triggered by the knockout of *gf14e*. Moreover, relative to the *dsGFP* group and untreated control, silencing *Nl14* significantly lowered the female adult mass, fecundity, and egg‐hatching rates of BPH on WT plants (Figure 6J‐L, left lane), but the performance of *dsNl14*‐BPH were significantly higher on the oeNl14 and oeGF14e plants than on the WT plants (Figure 6J‐L, right two lanes), suggesting that overexpression of OsGF14e can rescue the reduced insect performance caused by *Nl14* silencing. Taken together, the effector Nl14 mimics the function of the plant immune repressor OsGF14e.

**Figure 7:**
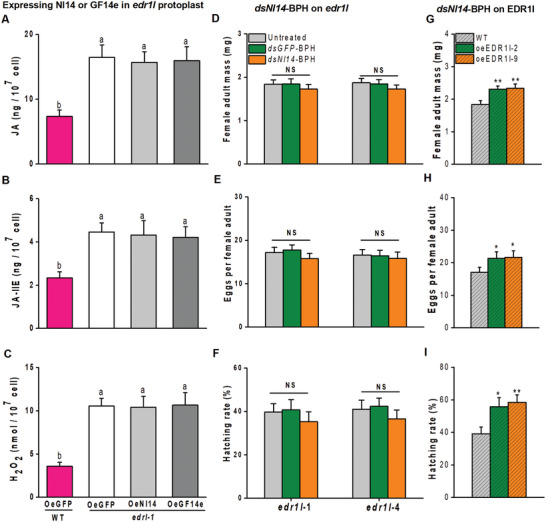
Nl14 suppresses rice defense responses through OsEDR1l. A‐C) Mean levels ± SEM (*n* = 6) of JA, JA‐Ile, and H_2_O_2_ in the protoplasts of *edr1l* mutant and WT rice plants. Nl14‐GFP (oeNl14) or GF14e‐GFP (oeGF14e) was transiently expressed in rice protoplasts via polyethylene glycol‐mediated transfection transformation. Expressing GFP (oeGFP) served as a vector control. D‐F) Mean mass ± SEM (*n* = 10) of a newly emerged female adult (D), mean number ± SEM (*n* = 9–12) of eggs (E) and mean eggs hatching rate ± SEM (*n* = 9–15) (F) of untreated BPH or *dsNl14*‐BPH or *dsGFP*‐BPH on *edr1l* mutant lines. G‐I) Mean mass ± SEM (*n* = 10) of a newly emerged female adult (G), mean number ± SEM (*n* = 9–12) of eggs (H) and mean eggs hatching rate ± SEM (*n* = 9–15) (I) of *dsNl14*‐BPH on the oeEDR1l lines or WT plants. Asterisks indicate significant differences between transgenic and WT plants (*, *p* < 0.05; **, *p* < 0.01; Student's *t*‐test). Different letters indicate a significant difference among treatments (*P* < 0.05; NS, not significant, one‐way ANOVA followed by Duncan's multiple range test). Error bars represent standard errors.

**Figure 8:**
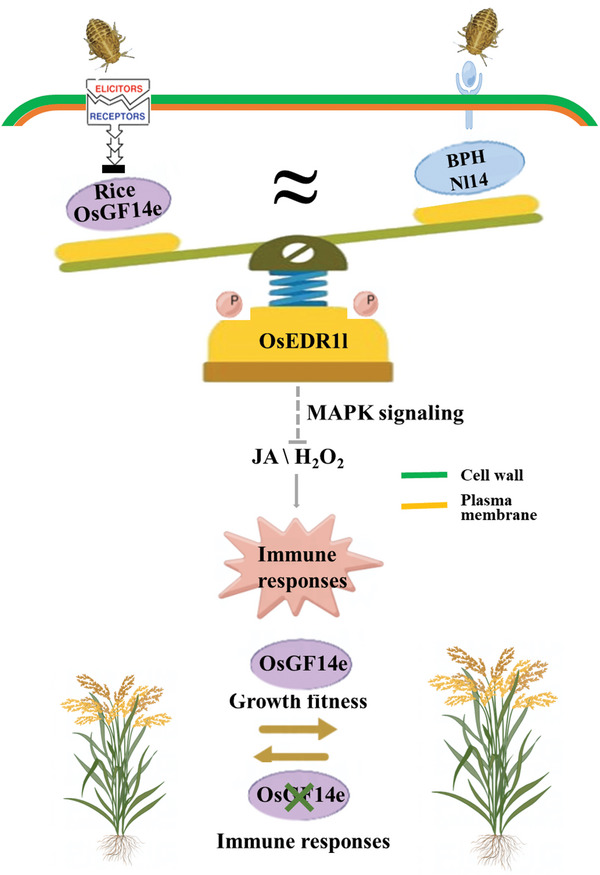
Proposed model for mimicry of rice immune repressor OsGF14e by BPH effector N114. The rice OsGF14e‐EDR1l module fine‐tunes immune activation, thus ensuring a balance between defense and plant growth. Under normal conditions, high OsGF14e expression recruits OsEDR1l and enhances OsEDR1l‐mediated MAPK cascade to suppress JA and H_2_O_2_ accumulation, which restricts immunity and promotes plant growth and grain production. Upon insect infestation, the OsGF14e‐OsEDR1l module is suppressed through the spatiotemporally limited downregulation of *OsGF14e* expression, and the OsGF14e signaling circuit is rewired towards active immune responses that confer resistance to BPH at the expense of growth. Intriguingly, the BPH effector Nl14 shares high sequence homology and structural similarity with OsGF14e and thus executes a function similar to that of OsGF14e. Nl14 from BPH saliva and egg‐associated secretions enters rice tissues during BPH feeding and oviposition, compensates for the decrease in OsGF14e regulated by BPH, and exploits the OsGF14e‐EDR1l immune suppression module to facilitate BPH infestation.

Furthermore, we investigated whether Nl14 suppresses rice defense responses through OsEDR1l. Overexpression Nl14 and OsGF14e suppressed the levels of JA, JA‐Ile, and H_2_O_2_ in WT plants or *gf14e* mutants’ protoplasts (Figures 1C–E and 6A–C,G–I), but overexpression of either Nl14 or OsGF14e in rice protoplasts was unable to suppress the defense signaling triggered by the loss of *edr1l* (**Figure** **7**A–C). Moreover, silencing *Nl14* significantly lowered the performance of BPH on WT plants (Figure 6J–L), but such reduction diminished when these BPHs were maintained on *edr1l* mutants (Figure 7D–F). This suggests that Nl14 does not exhibit effector function in the absence of OsEDR1l. To further confirm the role of OsEDR1l in mediating the effector function of Nl14, we compared the performance of *dsNl14*‐BPH on the WT and oeEDR1l plants. The female adult mass, fecundity, and egg‐hatching rate of *dsNl14*‐BPH were significantly higher on the oeEDR1l plants than on the WT plants (Figure 7G–I), suggesting that overexpression of OsEDR1l can rescue the reduced insect performance caused by *Nl14* silencing. Taken together, the effector appears to exploit the signaling pathway mediated by the rice OsGF14e‐EDR1 module to impair plant defense against BPH (**Figure** **8**).

### 2.6 14‐3‐3e Proteins from the Saliva of Whitefly and Spider Mite Also Function as Effectors

The 14‐3‐3e widely exists in eukaryotes, and the phylogenetic tree indicates their close evolutionary relation within Arthropoda (Figure S20, Supporting Information). Moreover, the 14‐3‐3e homologs have also been identified in the saliva of whitefly *Bemisia tabaci (B. tabaci, Bt14)* and spider mite *Tetranychus evansi (T. evansi, Te14).^[^* ^26^ *^,^* ^28^ *^]^ Thus, they* were individually expressed in *N. benthamiana* to investigate whether the two 14‐3‐3e molecules facilitate fecundity of herbivorous arthropods. *B. tabaci* feeding on *N. benthamiana* expressing *Bt14 and T. evansi* feeding on *N. benthamiana* expressing Te*14 both* displayed higher fecundity than those feeding on GFP‐expressing leaves (Figure S21, Supporting Information). Together, these results suggest that 14‐3‐3e may be a conserved effector in herbivorous arthropods that impairs plant defenses.

## 3 Discussion

Plants are challenged by numerous herbivorous arthropods in their natural habitats. Because immune activation imposes fitness costs on plant growth, an efficient and dynamic immune system must be maintained to balance between warding off potential herbivores and avoiding negative impacts on plant growth. Here, we showed that the immune repressor OsGF14e balances plant defense and growth. Under normal conditions without insect infestation, high expression of *OsGF14e* recruits OsEDR1l and enhances OsEDR1l‐mediated MAPK signaling, which restricts immunity and promotes plant growth. Upon BPH infestation, the OsGF14e‐OsEDR1l module is dynamically suppressed through the spatiotemporally limited downregulation of *OsGF14e* expression, thereby permitting JA, JA‐Ile, and H_2_O_2_ production to activate short‐term and local defense responses at the expense of plant growth. However, after 72 h of BPH infestation, the *OsGF14e* expression and its mediated plant defense return to the control level without insect infestation. This growth‐defense trade‐off might be attributed to crosstalk between JA and central growth‐related phytohormone gibberellin (GA). JA has been reported to play a key role in prioritizing defense over growth during BPH attack, because JA signaling simultaneously activates defense responses and GA catabolism to rapidly optimize resource allocation to defense in attacked plants.^[^ ^33^ ^]^ A decrease in GA levels negatively affects plant growth, resulting in dwarf plants.

Plants have developed complex and accurate mechanisms to balance defense and growth in the course of their long‐term coevolution with herbivorous insect. For instance, when BPH feeds on resistant rice plants carrying *Bph14*, the salivary effector BISP interacts with Bph14 to trigger resistance. However, this constitutive defense response is costly, as reflected in restricted rice growth and grain yield. Thus rice can degrade BISP to fine‐tune immune homeostasis through the autophagy pathway, and reset their immune systems to the “off ” status, thereby reallocating resources to growth and reproduction.^[^ ^8^ ^]^ In addition, the transcript levels of immune regulators are dynamically regulated in plant response to herbivorous insects. The expression pattern of *OsGF14e* was reminiscent of that of the DELLA gene *OsSLR1*, which also negatively regulates rice defense against BPH. *OsSLR1* is downregulated by BPH infestation in the early stage but shows no difference from uninfested plants after 24 h. However, mechanical wounding only slightly altered *OsGF14e* and *OsSLR1* transcript abundance,^[^ ^34^ ^]^ suggesting that the expression pattern of some immune repressors is conserved. Thus, immune regulators, such as OsGF14e and OsSLR1, may be regulated by components in the saliva or egg‐associated secretions of BPH. However, the detailed mechanisms of *OsGF14e* regulation by BPH infestation require further investigation.

14‐3‐3 proteins are regulators (chaperones, activators, and repressors) of plant immunity. For example, several 14‐3‐3 proteins are positive regulators of disease resistance in *Arabidopsis*, *S. lycopersicum*, and *N. benthamiana* by interacting with MAPKKK and activating MAPK cascades.^[^ ^14^, ^15^ ^]^ In rice, the 14‐3‐3 protein GF14c also positively regulates plant disease resistance by modulating the homeostasis of a GRAS protein.^[^ ^35^ ^]^ In contrast, rice OsGF14e negatively regulates cell death and ROS burst, and impairs resistance to the bacterial blight pathogen Xoo and necrotrophic sheath blight pathogen *Rhizoctonia solani*;^[^ ^31^ ^]^ however, the target protein interactions with OsGF14e were not identified. Similar to that observed for the role of OsGF14e in rice‐pathogen interactions, OsGF14e impaired rice resistance against BPH. Apparently, 14‐3‐3 proteins have different immunity regulatory functions in various plant species. However, for the first time, we identified the target protein of OsGF14e, and provided direct evidence that rice 14‐3‐3 proteins regulate plant immunity by interacting with MAPKKK and enhancing its protein abundance as well. It has been reported that one role of 14‐3‐3 proteins is to stabilize their target proteins by preventing their accessibility to proteases. ^[^ ^12^, ^14^ ^]^ For example, 14‐3‐3 proteins enhance the stability of the phosphorylated microtubule‐severing enzyme spastin by inhibiting the ubiquitin‐proteasome pathway. ^[^ ^36^ ^]^ Here, we observed that OsGF14e did not affect the transcript levels of OsEDR1l, but it increased the protein abundance of OsEDR1l. Moreover, treatment with the proteasome inhibitor MG132 abolished the observed difference in OsEDR1l accumulation. This suggests that OsGF14e binding may promote the OsEDR1l accumulation, possibly by preventing its ubiquitin‐mediated degradation. The finding that OsGF14e negatively regulates broad‐spectrum diseases and insect resistance provides insights for the development of new strategies for rice pest control.

During feeding and oviposition, herbivorous insects secrete hundreds of proteins into plant tissues. For instance, during BPH stylet penetration of rice cell walls, salivary proteins are secreted directly into plant cells. During oviposition, the BPH ovipositor cuts through the rice leaf sheath, depositing eggs within and creating numerous wounds.^[^ ^30^ ^]^ The fluids surround the eggs are also released into the damaged plant tissue. Thus, the proteins in BPH egg‐associated secretions likely enter plant cells via endocytosis, similar to the feeding mechanism of chewing insects.^[^ ^37^ ^]^ Different effectors from a species exert diversified roles in insect‐plant interactions. Some salivary proteins activate plant defense. However, most of the known egg‐associated elicitors of plant defenses are low‐molecular‐ weight organic compounds.^[^ ^38^, ^39^, ^40^ ^]^ Only 2 proteinaceous elicitors, annexin‐like protein from sawfly *Diprion pini* ^[^ ^41^ ^]^ and small N‐terminal subunit of vitellogenin from BPH, ^[^ ^42^ ^]^ have only recently been characterized in activating plant defense. While others, particularly salivary proteins, exert multiple roles in impairing plant defense, such as calcium‐binding proteins for calcium signaling regulation, ^[^ ^43^ ^]^ endo‐β‐1, 4‐glucanase for cellulose degradation,^[^ ^44^ ^]^ and salivary sheath proteins interaction in rice planthoppers. ^[^ ^45^ ^]^ Insect egg‐ associated secretions play important roles in facilitating egg laying and hatching,^[^ ^30^ ^]^ but from which individual effector except Nl14 that suppresses plant defense has not been identified.

To maintain insect feeding and oviposition, the production of anti‐insect signalings must be tightly suppressed,^[^ ^4^, ^45^ ^]^ thus revealing a potential point of interference for herbivore effectors in the attenuation of OsGF14e downregulation‐mediated defenses. Pathogens, in turn, have evolved mechanisms that mimic host plant cellular process regulators to exploit host immune modules. For example, the bacterial effector HopBF1 structurally mimics the host HSP90 client to facilitate pathogenicity.^[^ ^46^ ^]^ The fungal effector AvrPiz‐t structurally mimics the host ROD1 and shares the same ROS‐elimination cascade to suppress rice immunity and promote fungi virulence.^[^ ^7^ ^]^ The effector of the oomycete *Phytophthora palmivora* mimics a 14‐3‐3‐binding motif to promote its infection.^[^ ^47^ ^]^ Our study indicated that herbivores have adopted a similar approach to pathogens in suppressing host immunity by effector mimicking a key immune regulator. The secretion of effector Nl14 into rice tissue could compensate for the BPH infestation‐induced decrease of OsGF14e in rice and exploit the OsGF14e‐EDR1l immune suppression module to promote insect infestation. To the best of our knowledge, no such effector mimicry has been previously reported in herbivorous arthropods. Therefore, our study provides a new example of cross‐kingdom convergence on the regulation of JA, JA‐Ile, and H_2_O_2_ production, thus revealing an additional arena in the arms race between plants and insects.

In summary, the immune suppression module OsGF14e‐EDR1l balances plant immunity and growth through the dynamic regulation of *OsGF14e* expression, but it can be exploited by the BPH effector Nl14. Thus, BPH may have evolved a mechanism to attack rice by mimicking key regulators in the plant's defensive machinery. Our study reveals a previously unrecognized mechanism of host‐insect functional commonality in plant immune regulation, which may help explain the extraordinary success and impact of BPH as a major rice pest.

## 4 Experimental Section

### Plant Growth and Insect Rearing

The japonica rice *Oryza sativa* variety, *Nipponbare*, was used as the WT plant. Pre‐germinated seeds were cultured in plastic bottles in a greenhouse at 28 ± 2 °C under a 14 h: 10 h, light: dark photoperiod. Each 7‐day‐old seedling was transferred into a 750‐mL plastic pot containing potting medium. After 30 days, individual rice plants were ready for use in subsequent experiments. The *N. benthamiana* was grown in a climate chamber at 23 ± 1 °C under a 16 h: 8 h, light: dark photoperiod. After 4–5 weeks (five‐leaf stage), these tobacco plants were used in subsequent *Agrobacterium tumefaciens* (*A. tumefaciens*)‐mediated transient transformation experiments. Original colonies of BPH were obtained from rice fields in Nanjing, China, and maintained on rice seedlings in a climate chamber at 28 ± 1 °C under a 14 h: 10 h, light: dark photoperiod.

### Three‐Dimensional Structures Modeling

The *OsGF14e* and *Nl14* sequences were predicted from their genomic data. First, the α‐helices, β‐folds, loop regions, disordered domains, and transmembrane regions within the secondary structures of OsGF14e and Nl14 proteins were predicted according to the protein secondary structure analysis workstation (http://bioinf.cs.ucl.ac.uk/psipred/). Subsequently, the AlphaFold program was used to model the 3D structure of proteins from scratch.^[^ ^48^ ^]^ The PROCHECK program was used to evaluate the complete and optimized protein model.^[^ ^49^ ^]^

### Vector Construction

The In‐Fusion HD cloning kit (TaKaRa, Otsu, Japan) was used to insert *Nl14* or *GF14e* into the subcellular localization vector *pBINPLUS‐GFP* (*GFP*), Co‐IP vector *pBINPLUS‐mCherry* (*mCherry*), Y2H vector *pGBKT7* (*BD*) and BiFC vector *PCV‐YFP‐C* (*cYFP*). Similarly, OsEDR1l and its homologous genes were cloned into the subcellular localization and Co‐IP vector *GFP*, Y2H vector *pGADT7* (*AD*), and BiFC vector *PCV‐YFP‐N* (*nYFP*), respectively. Each plant expression vector was controlled by the CaMV 35S promoter. The primers used for genes cloning are listed in Table S2 (Supporting Information).

### Transient Expression of the OsGF14e, Nl14, and OsEDR1l in *N. benthamiana*

The empty vector *GFP* or *mcherry* and the recombinant plasmid harboring *OsGF14e*, *Nl14*, or *OsEDR1l* were transformed by electroporation into *A. tumefaciens* strain GV3101, respectively. *A. tumefaciens* cultures were centrifuged and pellets were then resuspended in the infiltration buffer (10 mm MgCl_2_, 10 mm 2‐(N‐Morpholino) ethane sulfonic acid (MES), and 100 µm acetosyringone) to a final OD_600_ (optical density at 600 nm) of 0.6. The *A. tumefaciens* suspension was infiltrated into *N. benthamiana* leaves using a needleless syringe. Expression of GFP and mcherry was examined under an LSM750 confocal laser‐scanning microscope (Zeiss, Oberkochen, BW, Germany). After 12–24 h, an elicitor chitosan (0.04%, w/v) or a proteasome inhibitor MG132 (carbobenzoxyl‐l‐leucyl‐l‐leucyl‐l‐leucine, 50 µmol L^−1^) (MCE, New Jersey, NJ, USA) was injected at the same region that had been infiltrated. After 24 h, samples were collected for protein extraction.

### Generation and Characterization of Genome‐Edited Plants

The target sequences of *OsGF14e* or *OsEDR1l* were introduced into a *pBGK032‐OsU6b* to yield rice *U6b* promoter‐driven single‐guide RNA (*sgRNA*). The *sgRNA* expression cassette was then introduced into the plant CRISPR‐Cas9 binary vector *pYLCRISPR/Cas9Pubi‐H*.^[^ ^50^ ^]^ Two different targets of each gene were used, and T‐DNA was inserted into the *Nipponbare* genetic background using *A. tumefaciens*‐mediated transformation. The homozygous mutant plants were screened using hygromycin gene identification and DNA sequencing. To generate the overexpression lines, the full‐length ORFs of *OsGF14e*, *OsEDR1l*, or *Nl14* were ligated into vector pCAMBIA1301 under the control of the CaMV 35S promoter. Each T‐DNA was inserted into the genome of *Nipponbare* using *A. tumefaciens*‐mediated transformation. Homozygous T_2_ plants were selected using hygromycin resistance and GUS staining.

### JA, JA‐Ile, SA, and H_2_O_2_ Analyses

The wild‐type (*WT*) and transgenic plants were randomly assigned to the BPH and control treatments. Leaf sheaths were harvested at 0, 8, and 24 h after infestation by 15 gravid BPH females, with the sample harvest time points determined based on previous results.^[^ ^4^, ^42^ ^]^ The JA, JA‐Ile, and SA in rice tissues were extracted with ethyl acetate spiked with labeled internal standards (^2^D_6_‐JA, ^2^D_6_‐JA‐Ile, and ^2^D^4^‐SA) following a previously described method and analyzed using a high‐performance liquid chromatography‐tandem mass spectrometer (HPLC–MS/MS).^[^ ^51^ ^]^ The H_2_O_2_ was extracted using a previously described method, and the concentration of H_2_O_2_ was determined using an Amplex‐Red Hydrogen Peroxide/Peroxidase Assay Kit (Invitrogen, Carlsbad, CA, USA) following the manufacturer's instructions.^[^ ^52^ ^]^ Each treatment was replicated six times.

### Yeast Two‐Hybrid Assay for Screening Target Proteins of OsGF14e

Y2H analysis was performed according to the Matchmaker Gold Yeast Two‐Hybrid System User Manual (Clontech, Mountain View, CA, USA). Briefly, *OsGF14e* was fused to the Gal4 BD as a bait plasmid, and the cDNA library from rice fed by BPH was fused to the Gal4 AD as a prey plasmid. They were then transformed into the yeast cells Y2H Gold and Y187 for the subsequent detection of potential interactions. A total of 1 × 10^6^ clones were screened. Blue colonies on the double dropout medium SD/‐Leu/‐Trp supplemented with X‐α‐Gal and Aureobasidin A (AbA) were transferred onto quadruple dropout medium SD/‐Ade/‐His/‐Leu/‐Trp/ supplemented with X‐α‐Gal and AbA to conduct the screening. Positive colonies were selected as templates, which was followed by PCR amplification and sequencing to detect the inserted fragments. The NCBI database was blast‐searched to identify candidate target genes.

### Protein Extraction and Western Blot Assays

Rabbit anti‐OsEDR1l and anti‐Nl14 polyclonal antibodies against the peptides CGPDEQEGSRRQVSN in OsEDR1l and EAEGEAEQKEQLQDVEDQDV in Nl14 were prepared by GenScript (GenScript Biotech, Nanjing, China). To collect egg‐associated secretions (EWS), eggs laid by gravid females within 12 h were carefully collected from rice leaf sheaths placed under an Olympus SZ51 microscope (Olympus, Tokyo, Japan). All intact eggs were surface‐washed with phosphate‐buffered saline (PBS) containing 1 mm phenylmethanesulfonyl fluoride (PMSF) for 3 min. The collected EWS was concentrated using a YM‐3 Microcon centrifugal filter device (Millipore, Billerica, MA, USA) with a membrane nominal molecular weight limit of 3000 Da.

To get the BPH female adults which can oviposit but not feed, the gravid females were anaesthetized using carbon dioxide (CO_2_), then carefully removed their stylets using dissecting forceps under an Olympus SZ51 microscope. One day after infestation by 50 newly emerged BPH female adults (feeding only) or 20 BPH gravid females without stylets (oviposition only), BPH eggs were carefully removed from the eggs‐laid leaf sheaths under the microscope, and the outer three leaf sheaths of each rice stem from independently infested plants or untreated controls were harvested. Total proteins were extracted from *N. benthamiana* and rice tissues using RIPA (radio immunoprecipitation assay) Lysis Buffer II (100 mg tissue mL^−1^ buffer) according to the manufacturer's instructions (Sangon Biotech, Shanghai, China). The extracts were centrifuged at 10 000 × *g* for 5 min at 4 °C, and the supernatant from each sample was collected and then concentrated using a YM‐3 centrifugal filter device.

For western blot assays, the total proteins in each supernatant were separated by 12% SDS‐PAGE (sodium dodecyl sulfate–polyacrylamide gel electrophoresis) (Genscript Biotech, Nanjing, China), and the blot was detected using anti‐GFP, anti‐mCherry (Abmart, Shanghai, China), anti‐Nl14, or anti‐OsEDR1l antibodies. Detection signals were visualized using Clarity Western ECL Substrate (Bio‐Rad, Hercules, CA, United States). The gel was stained with a coomassie brilliant blue (CBB) to monitor the amount of input protein.

### Detection of Protein–Protein Interactions

In the Y2H assay, the bait plasmids *BD‐Nl14* and *BD‐OsGF14e* were co‐transformed with the prey plasmid *AD‐OsEDR1l* into AH109. Transformants were cultured on the SD/−Leu/−Trp medium, and SD/−Ade/−His/−Leu/−Trp/X‐α‐Gal medium for blue color development.

For Co‐IP, the constructs *Nl14‐mCherry* (or *OsGF14e‐mCherry*) and *OsEDR1l‐GFP* were transformed into GV3101 and co‐infiltrated into *N. benthamiana* leaves. After 48 h, the total proteins in the injected leaves were incubated for 2 h with RFP‐Trap A beads (ChromoTek Martinsried, BA, Germany). After washing six times with wash buffer, the beads were boiled in 1× sodium dodecyl sulfate (SDS) loading buffer for 10 min. The protein samples were then subjected to western blotting analysis using anti‐GFP and anti‐mCherry.

For the BiFC assay, a previously published procedure was followed.^[^ ^4^ ^]^ Briefly, *cYFP‐Nl14* (or *cYFP‐GF14e*) and *nYFP‐OsEDR1l* were transformed into *Agrobacterium* strain GV3101, which was subsequently co‐infiltrated into *N. benthamiana* leaves. After 48 h, the YFP signals (514 nm) were observed using an LSM750 confocal laser‐scanning microscope (Zeiss, Germany).

### Purification of Recombinant Protein

The *OsGF14e* and *Nl14* were fused in‐frame with the His tags in vector *pET32a*, and the *OsEDR1l* was fused in‐frame with the GST tag *pGEX‐6p‐1*, respectively. Primers used for vector construction were listed in Table S2 (Supporting Information). Subsequently, the expression of *His‐OsGF14e*, *His‐Nl14*, and *GST‐OsEDR1l* in *E. Coli BL21* cells was induced by 0.5 mm isopropyl b‐d‐1‐thiogalactopyranoside (IPTG) overnight at 16 °C. The proteins were purified using HisSep Ni‐NTA MagBeads (Yeasen, Nanjing, China) and glutathione‐Sepharose 4B beads (GE Healthcare, Nanjing, China) according to the manufacturers’ instructions, respectively.

### In Vitro Analysis of OsEDR1l Phosphorylation Level

Purified 1 µg OsEDR1l protein was mixed with purified 1 µg OsGF14e or Nl14 in a 25 µL reaction buffer containing 25 mm Tris–HCl (pH 7.5), 10 mm MgCl_2_, 100 mm CaCl_2_ 1 mm DTT, and 100 µm ATP at 37 °C for 45 min, respectively. The reaction buffer contained only purified OsEDR1l, OsGF14e, or Nl14 were used as control. After reaction, ATP and other salt ions were removed from the reaction system by using Sephadex DeSalting Gravity Column (Sangon Biotech, Shanghai, China) according to the instructions. Subsequently, the Phosphoprotein Phosphate Estimation Kit (Sangon Biotech, Shanghai, China) was used to detect the phosphorylation levels of the samples. Briefly, each 50 µL desalted sample was mixed with 50 µL of 2.0 mol/L NaOH, and incubated at 65 °C for 30 min; After the solution cooled to room temperature, 50 µL 4.7 mol L^−1^ HCl and 50 µL detection reagent were added to each volume and mixed well, then left to stand at room temperature for 30 min. Finally, the absorbance at A_620_ of each sample was measured using a microplate spectrophotometer (BioTek, Winooski, VT, USA), the concentration of sample phosphorylation was calculated using the standard curve. The data represents the percentage of moles of phosphorus per mole of phosphorylated protein.

### Phosphorylation Analysis of MAPKs

OsGF14e and Nl14 transgenic and WT plants samples were collected, and total proteins were extracted using RIPA Lysis Buffer II containing a phosphatase inhibitor (Sangon Biotech, Shanghai, China). The mixture was separated by 12% SDS‐PAGE, and the phosphorylated levels of OsMAPK3/6.blot were detected in western blot using Phospho‐p44/42 MAPK (Erk1/2) (Thr202/Tyr204) antibodies (Cell Signaling Technology, Lane Danvers, MA, USA).

### Protoplast Isolation and Transfection

Rice protoplasts were isolated and transfected as described previously.^[^ ^53^ ^]^ Briefly, 10‐day‐old transgenic rice or WT seedlings were used for protoplasts isolation. After the viability of protoplasts was determined by the fluorescein diacetate (FDA) staining method, the density of protoplasts was adjusted to 1×10^8^ mL^−1^ using a hemocytometer. PEG‐mediated transfections were carried out. A total of 15 µg plasmid DNA (*Nl14‐GFP* or *OsGF14e‐GFP*) was added to each sample mixed with 100 uL protoplasts (≈10^7^ cells) and 110 µL polyethylene glycol (PEG) solution. After 16 h incubation in darkness, green fluorescence was examined under an LSM750 confocal laser‐scanning microscope (Zeiss, Germany). Finally, the protoplasts were harvested by centrifugation for JA, JA‐Ile, SA, and H_2_O_2_ analysis.

### Sequence Analysis and Phylogenetic Tree Construction

The domains and nuclear localization sequences of OsEDR1l were analyzed using ScanProsite program from Expasy server (au.expasy.org/prosite/) and NLStradamus online software (moseslab.csb.utoronto.ca/NLStradamus/), respectively. To investigate the evolutionary relationship among 14‐3‐3e members from Arthropoda and MAPKKK proteins from rice, phylogenetic trees were constructed by employing the minimal evolution (ME) method and the neighbor‐joining (NJ) method wrapped in MEGA11 software suite.

### Plant Treatments

For mechanical wounding, plants (one per pot) were individually damaged by creating 100 holes (wound) with an insect pin (diameter 0.27 mm; Aladdin, Shanghai, China) on the lower part of the stems (≈2 cm long). The control plants were not pierced. For BPH treatment, plants (one per pot) were individually infested with 15 BPH gravid female adults or 25 BPH 5th instar nymphs that were confined in a glass cage (diameter 4 cm, height 8 cm) with small holes. Plants in empty cages served as controls.

### BPH Bioassays

BPH performance on different rice lines was evaluated based on female adult body mass, fecundity, and hatching rate of eggs. Twenty newly hatched nymphs were reared on each plant of different rice lines. Then, the mass of each group containing five female adults at 1 day after emergence was weighed (to an accuracy of 0.1 mg), with 10 biological replicates performed. For BPH fecundity and egg hatching rate, four days after emergence, 10 gravid females were confined in a glass cage and fed on the rice line corresponding to the one on which the nymphs had fed. After 24 h, the eggs were counted using a microscope. For egg hatching rate, one week after oviposition, the newly hatched nymphs were counted daily until no new nymphs were found. Unhatched eggs from oviposited leaf sheaths were counted under a microscope. Nine to fifteen biological replicates were performed. Each assay was repeated three times.

In the plant damage tests, transgenic line and WT plants were individually planted for ≈1 month. All pots of the tested transgenic lines and WT plants were placed in parallel in a cage and fed to the BPH population, which included 15 gravid BPH females per plant. The plant status (alive or dead) was surveyed daily the following week. Pictures of the status of each plant were captured when either of the tested lines or the control died. The experiment was repeated five times.

### *Bemisia tabaci* and *Tetranychus evansi* Bioassays

*Bt14* from *B. tabaci* Mediterranean type and *Te14* from *T. evansi* were transiently expressed in *N. benthamiana* leaves.^[^ ^26^, ^28^ ^]^ The bioassays were performed 48 h after *A. tumefaciens* infiltration of *N. benthamiana* leaves. For *B. tabaci* bioassay, Clip cages were fixed on the abaxial surface of the infiltrated leaf areas, and two female adults (2 days after reaching the adult stage) were released into each clip cage.^[^ ^54^ ^]^ After 3 days, the number of eggs laid on the leaves within the clip cages was counted to assess whitefly performance. One clip cage per plant was set up and each clip cage was treated as a biological replicate, with 10 replicates for each treatment. The experiment was repeated three times. For *T. evansi* bioassay, Leaf discs (18 mm in diameter) were collected from the infiltrated leaves. One female (2 days after reaching the adult stage) was placed on each leaf disc, and the number of eggs was counted 4 days after the introduction of the mite using a microscope.^[^ ^55^ ^]^ Each leaf disc was a biological replicate, with 10 replicates per treatment. The experiment was repeated three times.

### Agronomic Performance Test

To investigate the agronomic performance of the transgenic rice plants, plants were grown in a field in Nanjing, China, and subjected to routine management. At harvest, 1000‐grain weight and grain yield per plant were recorded. The experiment was repeated 15 times.

### Statistical Analysis

Data were presented as means ± SEM. Differences among treatments were analyzed with one‐way ANOVA followed by Duncan's multiple range test to compare treatments (or a Student's *t*‐test when only two treatments were compared). Data analyses were performed using SPSS STATISTICS 19. For each assay, at least three biological replicates were recorded for each data point, and two or three independent experiments were performed.

## Conflict of Interest

The authors declare no conflict of interest.

## Author Contributions

R.J., J.M.F., and J.C.F. designed the research. J.M.F., R.J., S.L., J.L., J.L., Z.C.Z., X.Y.T., and S.Y. performed the experiments and analyzed and interpreted the data. M.F.J. provided valuable suggestions for the research. R.J. and J.M.F. wrote the manuscript. R.J., K.Y.Z., and J.C.F. revised the manuscript. All authors reviewed the manuscript.

## Supporting information



Supporting Information

## Data Availability

Sequence data are available in NCBI under the following accession numbers: OsGF14e (LOC_Os02g36974; XM_015770741), OsEDR1l (LOC_Os02g14530; XM_015770804.2), Nl14 (XM_022337835.2), Bt14 (XM_019046102.1), and Te14 (NCKV01005351.1). The data that support the findings of this study are available from the corresponding author upon reasonable request.

## References

[1] M. Erb , P. Reymond , Annu, Rev. Plant Biol. 2019, 70, 527. 30786233 10.1146/annurev-arplant-050718-095910

[2] M. C. Schuman , I. T. Baldwin , Rev. Plant Biol. 2016, 61, 373. 10.1146/annurev-ento-010715-023851 26651543

[3] M. Y. Liu , G. J. Hong , H. J. Li , X. L. Bing , Y. M. Chen , X. F. Jing , J. Gershenzon , Y. G. Lou , I. T. Baldwin , R. Li , Proc. Natl. Acad. Sci. U. S. A. 2023, 120, 23. 10.1073/pnas.2305007120 PMC10266023 37256931

[4] R. Ji , J. M. Fu , Y. Shi , J. Li , M. F. Jing , L. Wang , S. Y. Yang , T. Tian , L. H. Wang , J. F. Ju , H. F. Guo , B. Liu , D. L. Dou , A. A. Hoffmann , K. Zhu‐Salzman , J. C. Fang , New Phytol. 2021, 232, 2. 10.1111/nph.17620 34260062

[5] Y. W. Deng , Y. S. Ning , D. L. Yang , K. R. Zhai , G. L. Wang , Z. H. He , Mol. Plant. 2020, 13, 1402. 32979566 10.1016/j.molp.2020.09.018

[6] B. Huot , J. Yao , B. L. Montgomery , S. Y. He , Mol. Plant. 2014, 7, 1267. 24777989 10.1093/mp/ssu049 PMC4168297

[7] M. J. Gao , Y. He , X. Yin , X. B. Zhong , B. X. Yan , Y. Wu , J. Chen , X. Y. Li , K. R. Zhai , Y. F. Huang , X. Y. Gong , H. Z. Chang , S. H. Xie , J. Y. Liu , J. X. Yue , J. L. Xu , G. Q. Zhang , Y. W. Deng , E. T. Wang , D. Tharreau , G. L. Wang , W. B. Yang , Z. H. He , Cell. 2011, 184, 21. 10.1016/j.cell.2021.09.009 34597584

[8] J. P. Guo , H. Y. Wang , W. Guan , Q. Guo , J. Wang , J. Yang , Y. X. Peng , J. H. Shan , M. Y. Gao , S. J. Shi , X. X. Shangguan , B. F. Liu , S. L. Jing , J. Zhang , C. X. Xu , J. Huang , W. W. Rao , X. H. Zheng , D. Wu , C. Zhou , B. Du , R. Z. Chen , L. L. Zhu , Y. X. Zhu , L. L. Walling , Q. F. Zhang , G. C. He , Nature. 2023, 618, 7966. 10.1038/s41586-023-06197-z PMC10284691 37316670

[9] Z. H. Liao , L. Wang , C. Z. Li , M. J. Cao , J. N. Wang , Z. L. Yao , S. Y. Zhou , G. X. Zhou , D. Y. Zhang , Y. G. Lou , Plant Cell Environ. 2022, 45, 9. 10.1111/pce.14341 35538611

[10] Y. S. Ning , W. D. Liu , G. L. Wang , Trends Plant Sci. 2017, 22, 1069. 29037452 10.1016/j.tplants.2017.09.010

[11] A. L. Paul , F. C. Denison , E. R. Schultz , A. K. Zupanska , R. J. Front , Plant Sci. 2012, 3. 22934100 10.3389/fpls.2012.00190 PMC3422896

[12] D. L. Darling , J. Yingling , A. Wynshaw‐Boris , Curr. Top. Dev. Biol. 2005, 68, 281. 16125003 10.1016/S0070-2153(05)68010-6

[13] R. Lozano‐Durán , S. Robatzek , Mol. Plant Microbe Interact. 2015, 28, 511. 25584723 10.1094/MPMI-10-14-0322-CR

[14] C. S. Oh , K. F. Pedley , G. B. Martin , Plant Cell. 2010, 22, 260. 20061552 10.1105/tpc.109.070664 PMC2828692

[15] X. J. Dong , F. Feng , Y. J. Li , L. Li , S. Chen , J. M. Zhou , Plant Cell. 2023, 35, 6. 36943771 10.1093/plcell/koad088 PMC10226567

[16] Z. Y. Gao , D. L. Zhang , X. L. Wang , X. Zhang , Z. Y. Wen , Q. S. Zhang , D. W. Li , S. P. Dinesh‐Kumar , Y. L. Zhang , Nat Commun. 2022, 13, 1. 35132090 10.1038/s41467-022-28395-5 PMC8821596

[17] E. Seo , X. Yan , D. Choi , Mol. Plant Microbe Interact. 2022, 36, 3. 10.1094/MPMI-06-22-0135-R 36413345

[18] S. Deb , M. K. Gupta , H. K. Patel , R. V. Sonti , Mol. Plant. 2019, 20, 7. 10.1111/mpp.12807 PMC6856769 31094082

[19] R. Chaudhary , H. C. Peng , J. M. He , J. MacWilliams , M. Teixeira , T. Tsuchiya , Q. Chesnais , M. B. Mudgett , I. Kaloshian , New Phytol. 2019, 221, 1518. 30357852 10.1111/nph.15475

[20] M. M. Zhang , J. B. Su , Y. Zhang , J. Xu , S. Q. Zhang , Curr. Opin. Plant Biol. 2018, 45, 1. 29753266 10.1016/j.pbi.2018.04.012

[21] K. P. Rao , T. Richa , K. Kumar , B. Raghuram , A. K. Sinha , DNA Res. 2010, 17, 139. 20395279 10.1093/dnares/dsq011 PMC2885274

[22] Y. Takahashi , J. B. Zhang , P. K. Hsu , P. H. O. Ceciliato , L. Zhang , G. Dubeaux , S. Munemasa , C. N. Ge , Y. D. Zhao , F. Hauser , J. I. Schroeder , Nat. Commun. 2020, 11, 1. 31896774 10.1038/s41467-019-13875-y PMC6940395

[23] X. L. Shen , H. B. Liu , B. Yuan , X. H. Li , C. G. Xu , S. P. Wang , Plant Cell Environ. 2011, 34, 2 10.1111/j.1365-3040.2010.02219.x 20807375

[24] L. L. Wang , G. J. Xu , L. H. Li , M. Y. Ruan , A. Bennion , G. L. Wang , R. Li , S. H. Qu , Proc. Natl Acad. Sci. USA. 2023, 120, 13. 10.1073/pnas.2211102120 PMC10068787 36952381

[25] H. J. Guo , Y. J. Zhang , J. H. Tong , P. P. Ge , Q. Y. Wang , Z. H. Zhao , K. Zhu‐Salzman , S. A. Hogenhout , F. Ge , Y. C. Sun , Curr. Biol. 2020, 30, 4826. 33035482 10.1016/j.cub.2020.09.020

[26] H. J. Huang , Z. X. Ye , G. Lu , C. X. Zhang , J. P. Chen , J. M. Li , Insect Sci. 2020, 28, 1369. 32757245 10.1111/1744-7917.12856

[27] H. J. Huang , J. B. Lu , Q. Li , Y. Y. Bao , C. X. Zhang , J. Proteomics. 2017, 172, 25. 29109049 10.1016/j.jprot.2017.11.003

[28] H. J. Huang , J. R. Cui , L. Chen , Y. X. Zhu , X. Y. Hong , Proteomics. 2019, 19, 4. 10.1002/pmic.201800302 30520223

[29] J. M. Fu , Y. Shi , L. Wang , H. Zhang , J. Li , J. C. Fang , R. Ji , Front. Plant Sci. 2021, 11, 622513. 33537052 10.3389/fpls.2020.622513 PMC7848103

[30] S. L. Li , J. Li , X. Y. Tan , Z. C. Zhao , L. Jiang , A. A. Hoffmann , J. C. Fang , R. Ji , Insect Sci. 2023, 31, 1135. 38010047 10.1111/1744-7917.13303

[31] P. M. Manosalva , M. Bruce , J. E. Leach , Plant J. 2011, 68, 777. 21793954 10.1111/j.1365-313X.2011.04728.x

[32] J. Xu , X. J. Wang , H. Y. Zu , X. Zeng , I. T. Baldwin , Y. G. Lou , R. Li , New Phytol. 2021, 230, 4. 10.1111/nph.17251 33533489

[33] G. C. Jin , J. F. Qi , H. Y. Zu , S. T. Liu , J. Gershenzon , Y. G. Lou , I. T. Baldwin , R. Li , Plant Cell. 2023, 35, 3828. 37392473 10.1093/plcell/koad191 PMC10533328

[34] T. L. Zhang , W. W. Wang , T. T. Cao , R. Li , Y. G. Lou , Plant Cell Environ. 2017, 40, 10. 10.1111/pce.13012 28666057

[35] L. Lu , Z. J. Diao , D. W. Yang , X. Wang , X. X. Zheng , X. Q. Xiang , Y. P. Xiao , Z. W. Chen , W. Wang , Y. K. Wu , D. Z. Tang , S. P. Li , Plant Cell Environ. 2022, 45, 1065. 35129212 10.1111/pce.14278

[36] Q. L. Liu , H. Yang , J. X. Luo , C. Peng , K. Wang , G. W. Zhang , H. S. Lin , Z. S. Ji , ELife. 2024, 12, RP90184. 38231910 10.7554/eLife.90184 PMC10945579

[37] Z. W. Yan , F. Y. Chen , X. Zhang , W. J. Cai , C. Y. Chen , J. Liu , M. N. Wu , N. J. Liu , B. Ma , M. Y. Wang , D. Y. Chao , C. J. Gao , Y. B. Mao , Nat. Commun. 2023, 14, 1.36596776

[38] P. Reymond , Chimia. 2022, 76, 11. 10.2533/chimia.2022.914 38069786

[39] M. Hilker , H. Salem , N. E. Fatouros , Annu. Rev. Entomol. 2023, 68, 451. 36266253 10.1146/annurev-ento-120120-100746

[40] E. Stahl , T. Brillatz , E. F. Queiroz , L. Marcourt , A. Schmiesing , O. Hilfiker , I. Riezman , H. Riezman , J. L. Wolfender , P. Reymond , Elife. 2020, 9. 10.7554/eLife.60293 PMC7521926 32985977

[41] J. Hundacker , N. Bittner , C. Weise , G. Bröhan , M. Varama , M. Hilker , Plant Cell Environ. 2022, 45, 1033. 34713898 10.1111/pce.14211

[42] J. M. Zeng , W. F. Ye , W. H. Hu , X. C. Jin , P. Kuai , W. H. Xiao , Y. K. Jian , T. C. J. Turlings , Y. G. Lou , New Phytol. 2023, 238, 1230. 36740568 10.1111/nph.18791

[43] T. Tian , R. Ji , J. M. Fu , J. Li , L. Wang , H. Zhang , S. Y. Yang , W. F. Ye , J. C. Fang , K. Zhu‐Salzman , Pest. Manage. Sci. 2021, 77, 5. 10.1002/ps.6252 33421243

[44] R. Ji , W. F. Ye , H. D. Chen , J. M. Zeng , H. Li , H. X. Yu , J. C. Li , Y. G. Lou , Plant Physiol. 2017, 173, 1920. 28126846 10.1104/pp.16.01493 PMC5338667

[45] H. J. Huang , Y. Z. Wang , L. L. Li , H. B. Lu , J. B. Lu , X. Wang , Z. X. Ye , Z. L. Zhang , Y. J. He , G. Lu , J. C. Zhuo , Q. Z. Mao , Z. T. Sun , J. P. Chen , J. M. Li , C. X. Zhang , Nat. Commun. 2023, 14, 737. 36759625 10.1038/s41467-023-36403-5 PMC9911632

[46] V. A. Lopez , B. C. Park , D. Nowak , A. Sreelatha , P. Zembek , J. Fernandez , K. A. Servage , M. Gradowski , J. Hennig , D. R. Tomchick , K. Pawlowski , M. Krzymowska , V. S. Tagliabracci , Cell. 2019, 179, 205. 31522888 10.1016/j.cell.2019.08.020 PMC6754304

[47] E. Evangelisti , A. Guyon , L. Shenhav , S. Schornack , Mol. Plant‐Microbe Interact. 2023. 10.1094/MPMI-12-22-0251-R 36734982

[48] J. Jumper , R. Evans , A. Pritzel , T. Green , M. Figurnov , O. Ronneberger , K. Tunyasuvunakool , R. Bates , A. Zídek , A. Potapenko , A. Bridgland , C. Meyer , S. A. A. Kohl , A. J. Ballard , A. Cowie , B. Romera‐Paredes , S. Nikolov , R. Jain , J. Adler , T. Back , S. Petersen , D. Reiman , E. Clancy , M. Zielinski , M. Steinegger , M. Pacholska , T. Berghammer , S. Bodenstein , D. Silver , O. Vinyals , et al., Nature. 2021, 596, 583. 34265844 10.1038/s41586-021-03819-2 PMC8371605

[49] R. A. Laskowski , M. W. Macarthur , D. S. Moss , J. M. Thornton , J. Appl. Crystallogr. 1993, 26, 283.

[50] X. Ma , Q. Zhang , Q. Zhu , W. Liu , Y. Chen , R. Qiu , B. Wang , Z. Yang , H. Li , Y. Lin , Y. Xie , R. Shen , S. Chen , Z. Wang , Y. Chen , J. Guo , L. Chen , X. Zhao , Z. Dong , Y. G. Liu , Mol. Plant. 2015, 8, 1274. 25917172 10.1016/j.molp.2015.04.007

[51] C. A. M. R. Lu , M. Riemann , M. Cosme , L. Mène‐Saffrané , J. Massana , M. J. Stout , Y. Lou , J. Gershenzon , M. Erb , Plant Physiol. 2015, 167, 1100. 25627217 10.1104/pp.114.252700 PMC4348761

[52] Y. G. Lou , I. T. Baldwin , Plant Physiol. 2006, 140, 1126. 16461381 10.1104/pp.105.073700 PMC1400569

[53] S. D. Yoo , Y. H. Cho , J. Sheen , Nat. Protoc. 2007, 2, 1565. 17585298 10.1038/nprot.2007.199

[54] L. S. Zang , Y. Q. Liu , S. S. Liu , Chin. Bull. Entomol. 2005, 42, 329.

[55] C. A. Villarroel , W. Jonckheere , J. M. Alba , J. J. Glas , W. Dermauw , M. A. Haring , T. Van Leeuwen , R. C. Schuurink , M. R. Kant , Plant J. 2016, 86, 119. 26946468 10.1111/tpj.13152

