# Supplementary material for: An Insect Effector Mimics Its Host Immune Regulator to Undermine Plant Immunity
Source: Adv Sci (Weinh). 2025 Jan 23;12(11):2409186. doi: 10.1002/advs.202409186 (PMC11923970; doi:10.1002/advs.202409186)
Supplement: Supplementary file 1 — Supporting Information [file ADVS-12-2409186-s001.docx]

**Supporting Information**

**
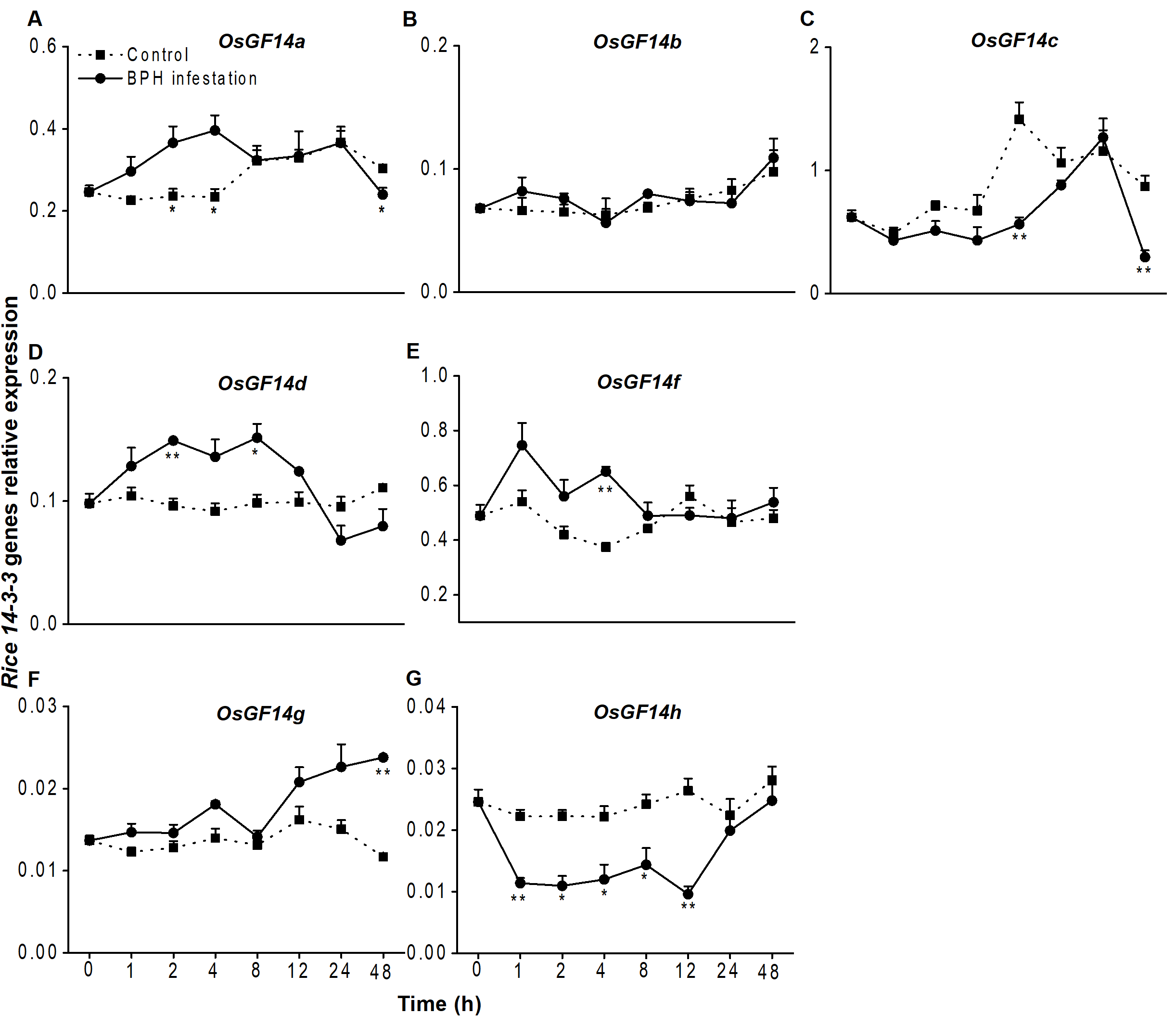
**

**Figure S1.** Expression pattern of *14-3-3* genes in rice response to BPH infestation. Mean transcript levels ± SEM (*n* = 3) of *14-3-3* genes in rice that were untreated or infested by BPH gravid female adults. Asterisks indicate significant differences between treatments and controls (*, *p* < 0.05; **, *p* < 0.01; Student’s *t*-test).

**
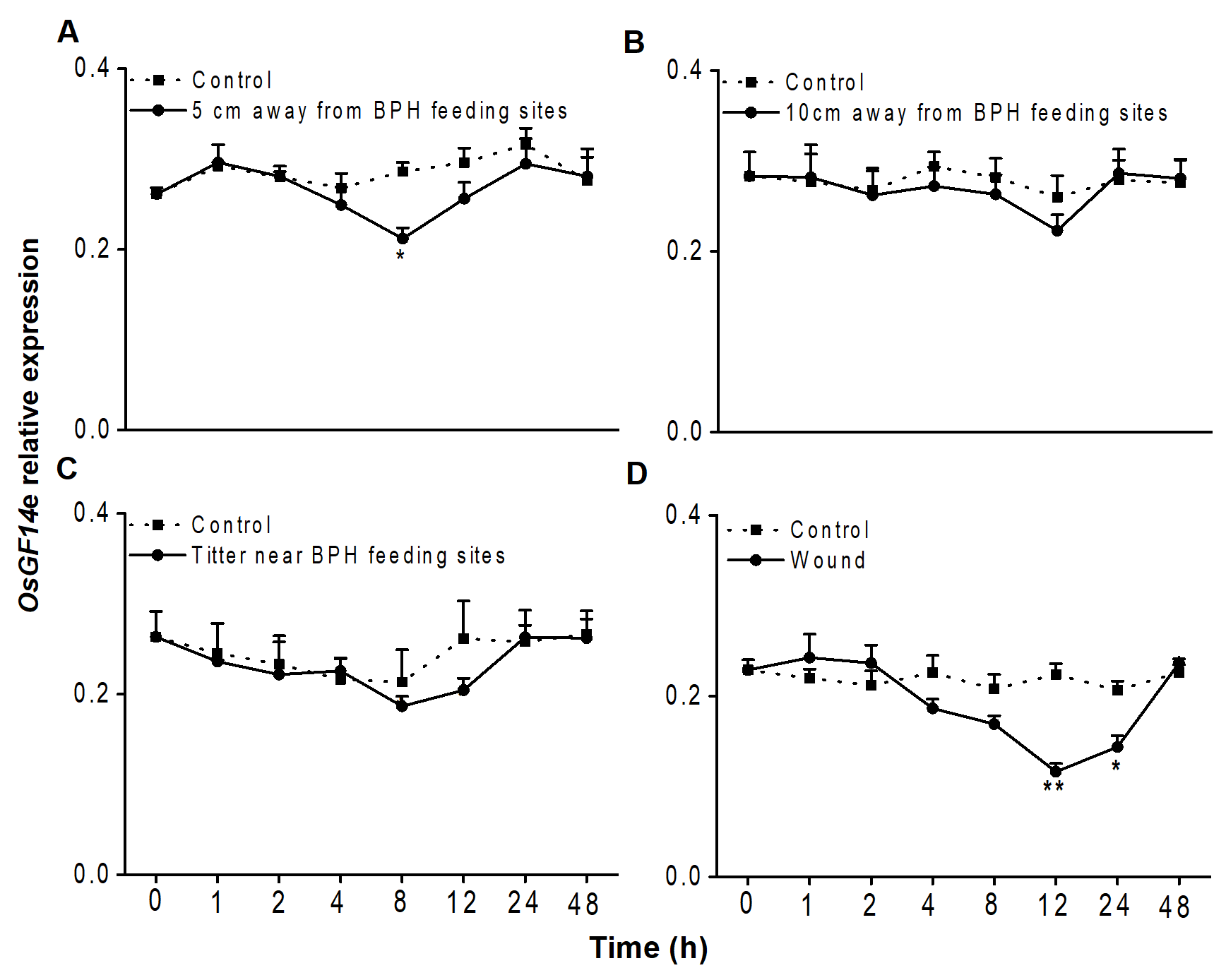
**

**Figure S2.** The regulation of *OsGF14e* in rice response to BPH infestation is local. A-B) Mean transcript levels ± SEM (*n* = 3) of *OsGF14e* in the area of leaf sheath 5 cm (A) or 10 cm (B) away near BPH feeding sites or the leaf sheath of the titer. C-D) closest to BPH feeding sites (C) or the leaf sheath mechanically wounded (D). Controls correspond to non-infested plants. Asterisk indicates significant difference (*, *p* < 0.05; **, *p* < 0.01; Student’s *t*-test).


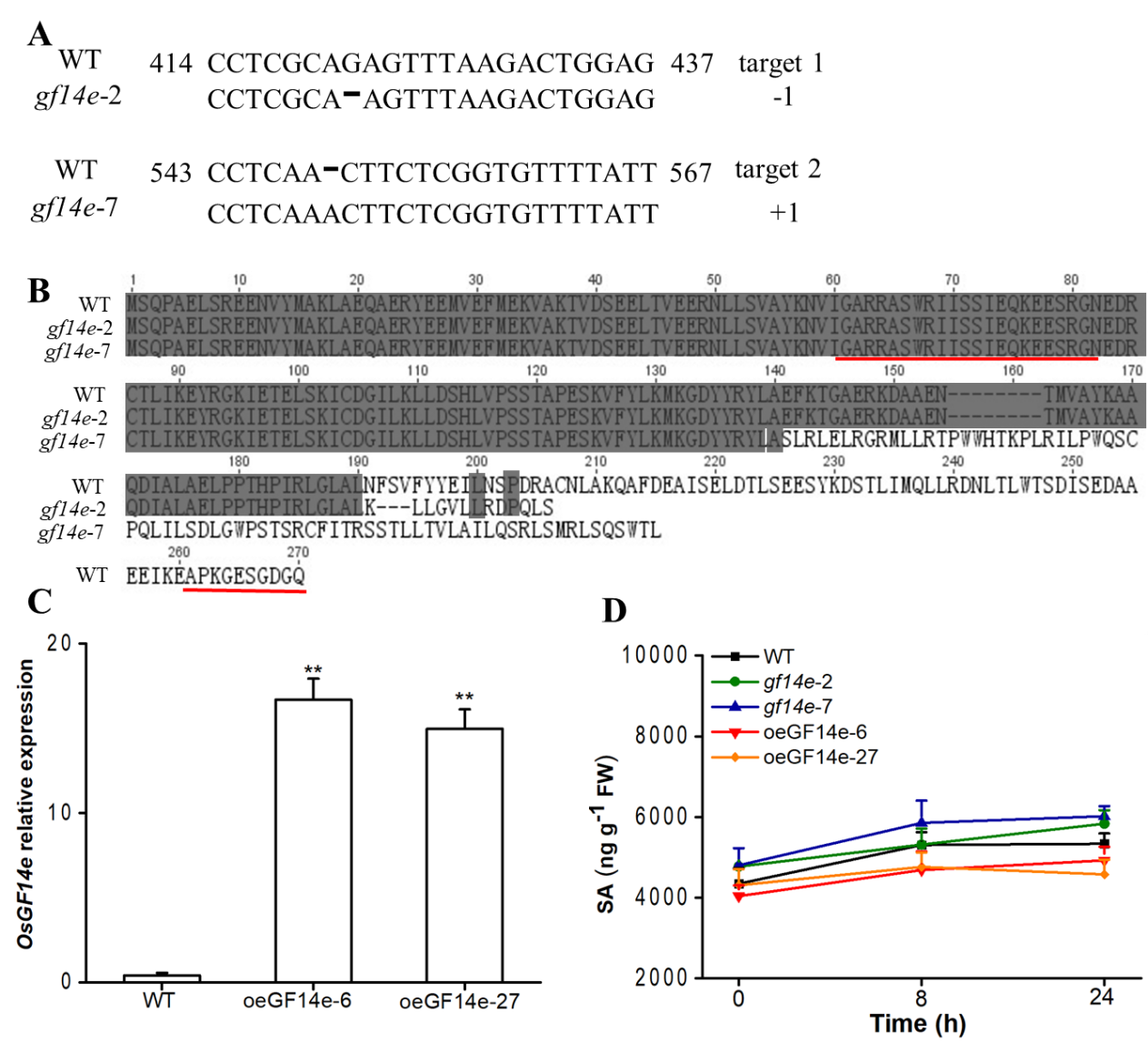


**Figure S3.** Mutation and overexpression of *OsGF14e* gene in rice. A) Mutation of *OsGF14e* gene by CRISPR-Cas9. The two target sequences of single-guide RNA (sgRNA) are shown. B) The amino acid sequence of oeGF14e in *gf14e* mutants and WT plants. The nuclear localization sequences are underlined in red. C) Mean transcript levels ± SEM (*n* = 3) of *OsGF14e* in WT and oeGF14e lines. Asterisks indicate significant differences between transgenic and WT plants (**, *p* < 0.01; Student’s *t*-test). D) Mean levels ± SEM (*n* = 6) of SA in *gf14e* mutants, oeGF14e and WT plants that were individually infested by BPH gravid female adults.


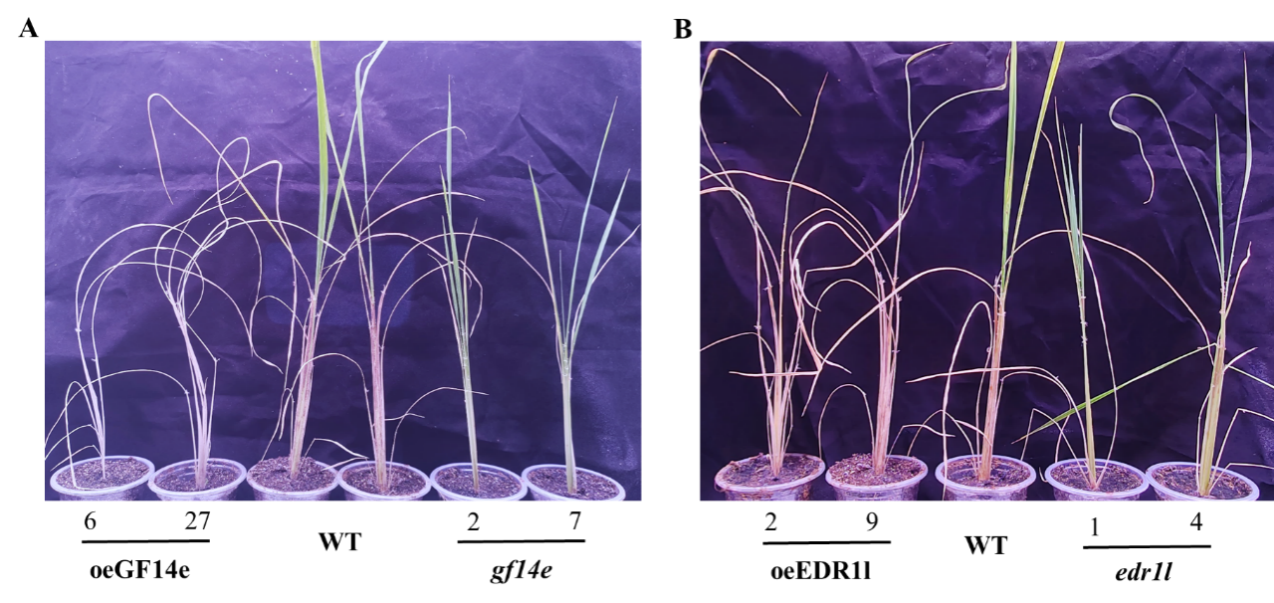


**Figure S4.** Damage phenotypes of transgenic lines and WT plants individually infested with BPH female adults for 12 days. The experiment was repeated five times, and the representative plants were showed.

**
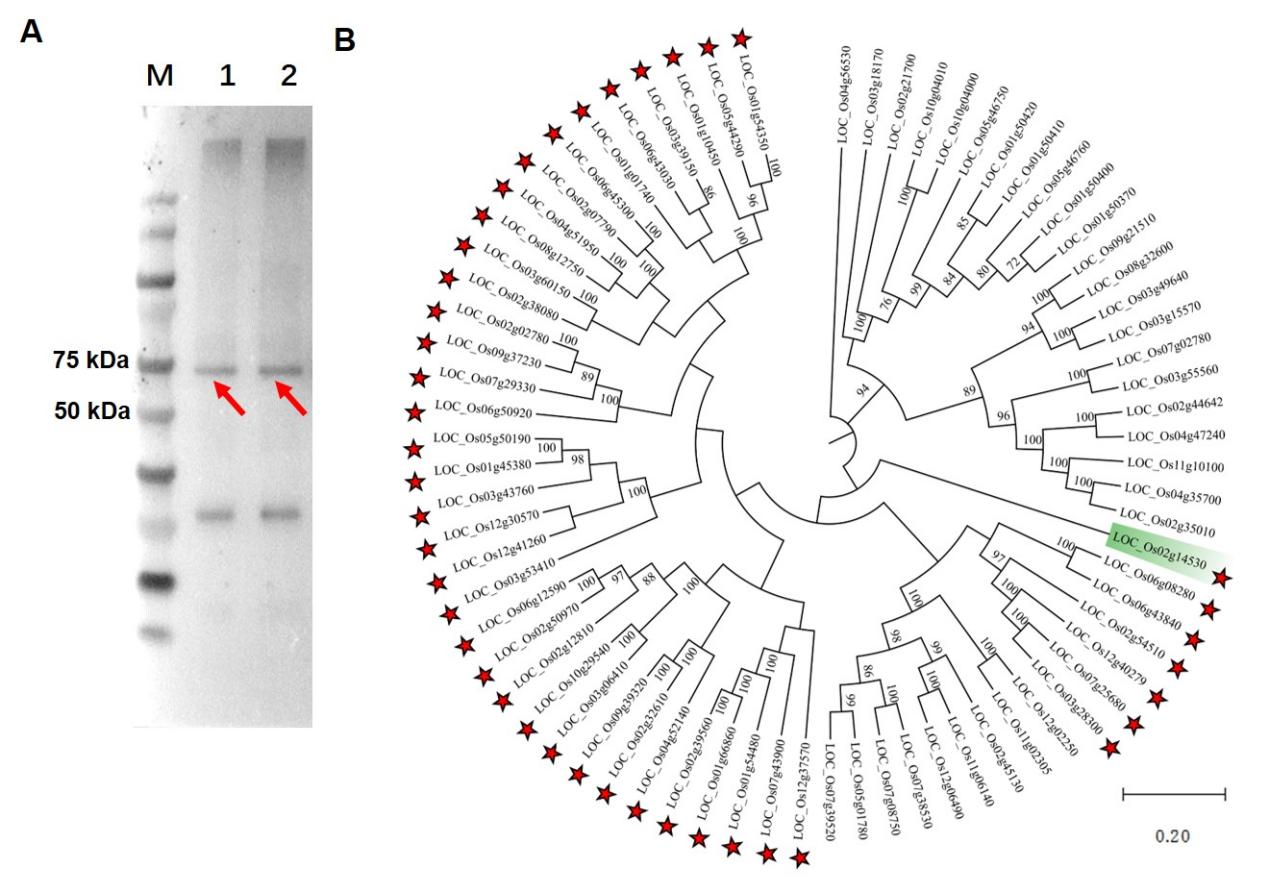
**

**Figure S5.** The analysis of MAPKKK members from rice. A) The analysis of OsEDR1l (74.48 kDa, arrowheads) in rice through western blot. M, marker; 1, *Nipponbare*; 2, ZH11. B) Phylogenetic relationships of 75 MAPKKK members from rice. The phylogenetic tree was constructed using MEGA 11.0 with Neighbor-Joining method. The scale bar represents 0.20 amino acid substitutions per site. Bootstrap values at phylogenetically are shown. Raf-like MAPKKKs are indicated by red stars. OsEDR1l (XM_015770804.2) in our study is shaded in green.


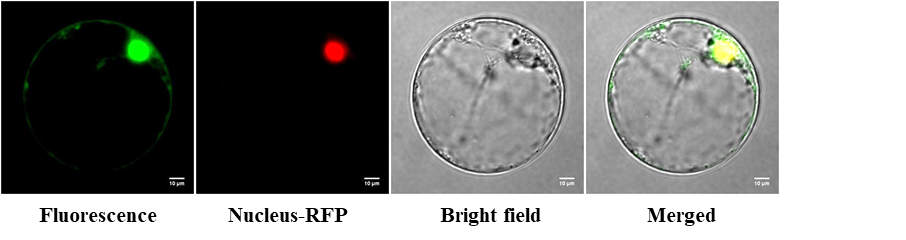


**Figure S6.** Cellular localization of OsEDR1l in rice protoplasts. OsEDR1l-GFP fusion proteins were expressed in rice protoplasts by polyethylene glycol-mediated transformation. Bars=10 μm.


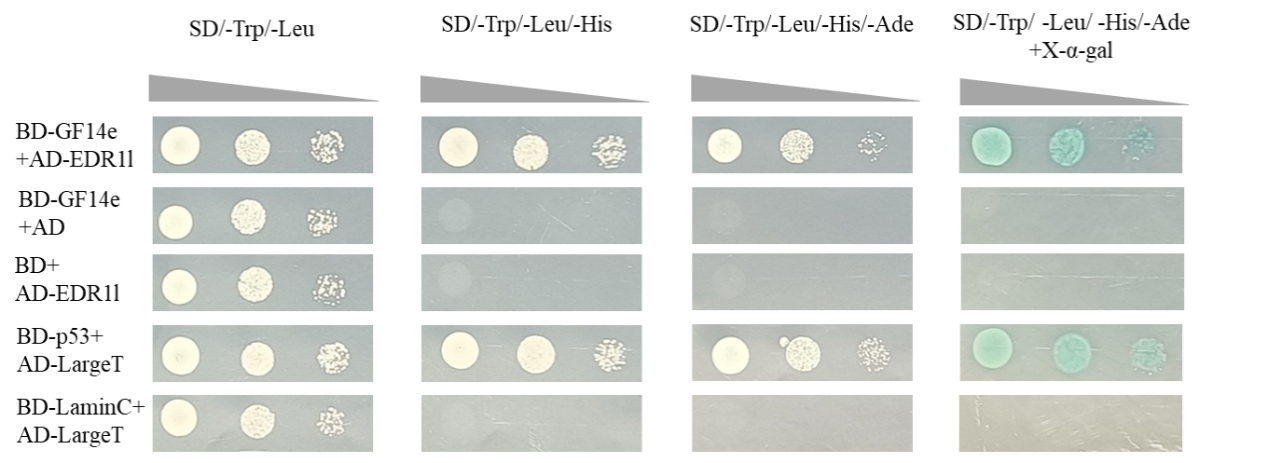


**Figure S7.** Y2H assays verifying interaction between OsGF14e and OsEDR1l. The growth of co-transformed Y2H Gold yeast colonies harboring indicated plasmids spotted on different nutrient-deficient SD/-Trp/-Leu, SD/-Trp/-Leu/-His, SD/-Trp/-Leu/-His/-Ade and SD/-Trp/-Leu/-His/-Ade medium with X-α-gal, respectively. BD-53 and AD-Large T works as a positive control and other combinations are as negative controls.

**
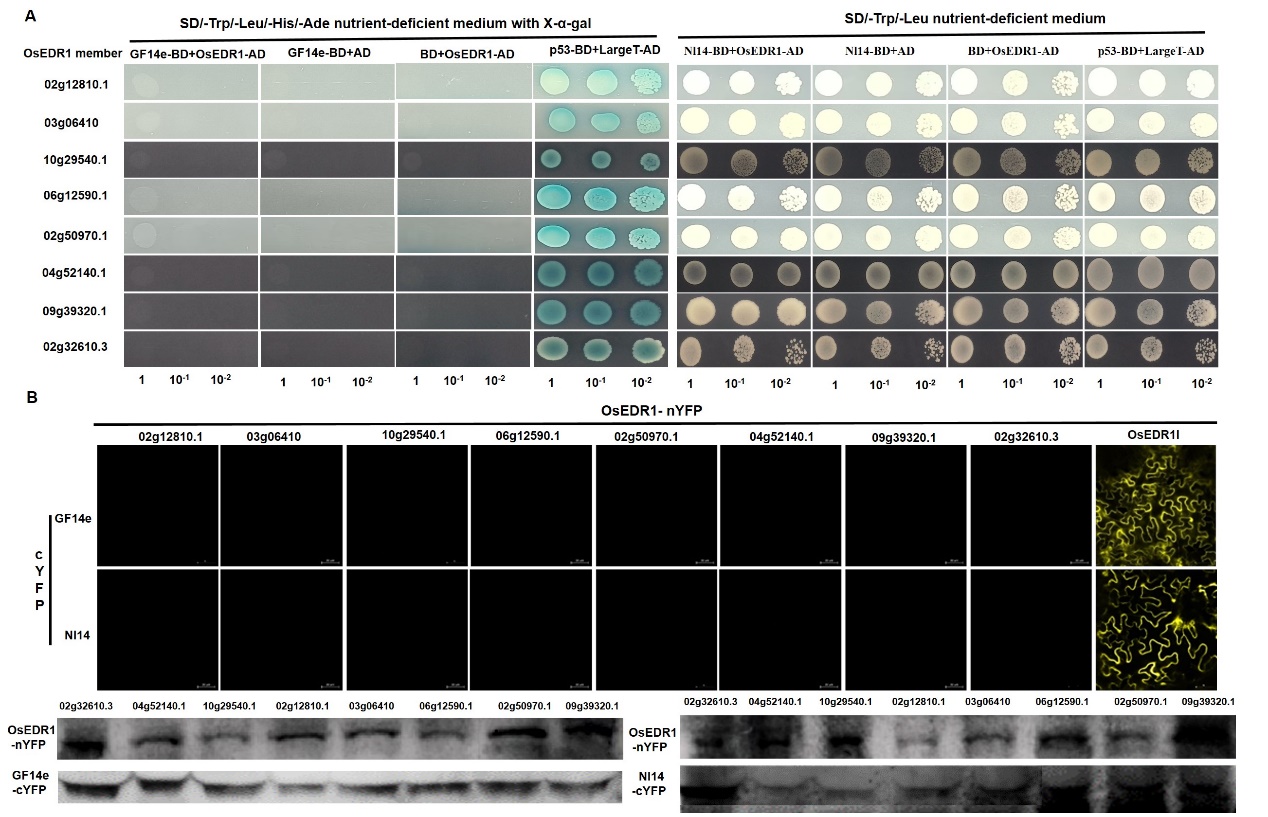
**

**Figure S8.** Interactions between OsGF14e or Nl14 and OsEDR1 members from rice. A) Y2H assay of yeast strain co-transformed with the indicated plasmids and spotted on the nutrient-deficient SD/-Trp/-Leu medium or SD/-Trp/-Leu/-His/-Ade medium with X-a-gal. BD-53 and AD-Large T works as a positive control. B) BiFC analysis to determine the interaction between OsGF14e or Nl14 and indicated OsEDR1 member in the leaf cells of *N. benthamiana*. Fluorescence indicates reconstitution of an intact fluorescent protein from the complementary OsGF14e-cYFP or OsNl14-cYFP and OsEDR1l-nYFP. Expression of the different recombinant plasmid in *N. benthamiana* was validated by western blot analysis (lower panel) using an anti-GFP antibody at 48 h post agro-infiltration.


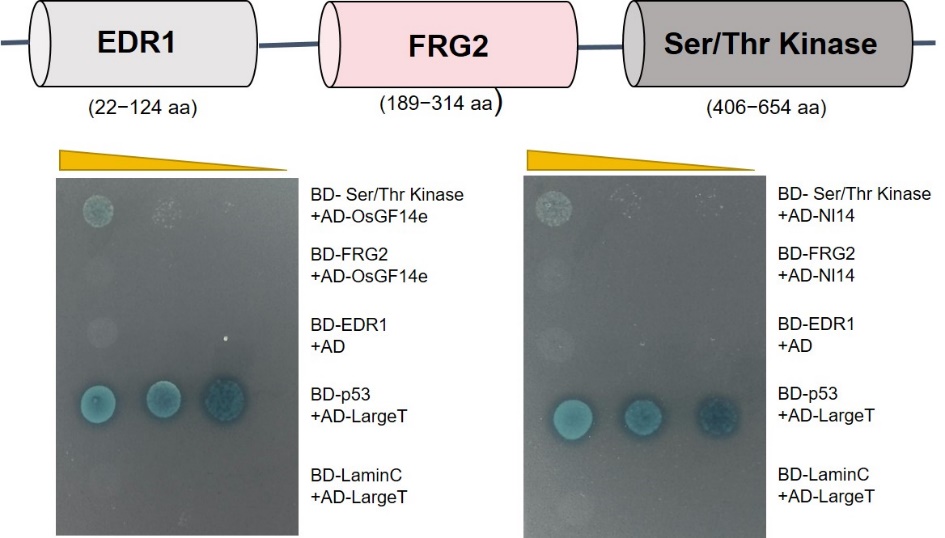


**Figure S9.** Interactions between OsGF14e or Nl14 and OsEDR1l domains. Y2H assay of yeast strain co-transformed with the indicated plasmids and spotted on the nutrient-deficient SD/-Trp/-Leu/-His/-Ade medium with X-a-gal. BD-53 and AD-Large T works as a positive control. BD-Lamin C and AD-Large T works as a negative control. FRG2, fascioscapulohumeral muscular dystrophy (FSHD) region gene 2.


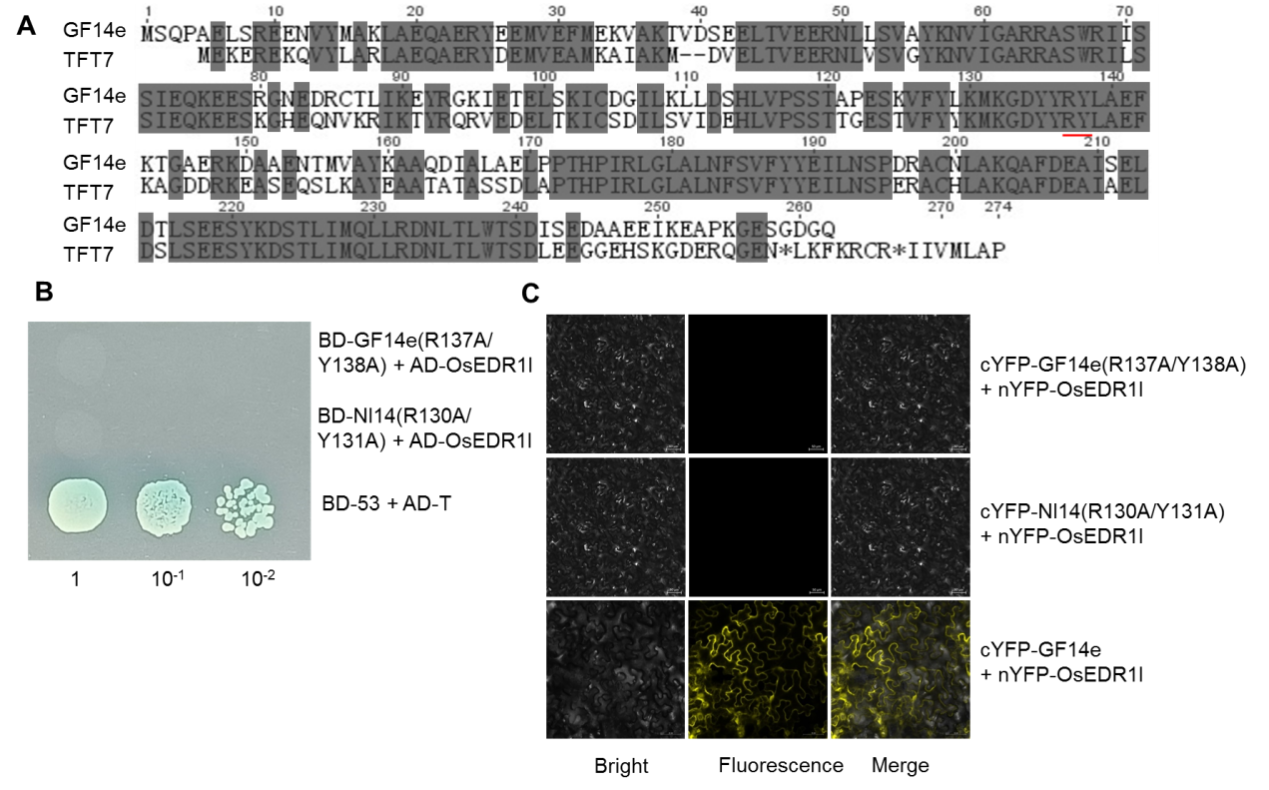


**Figure S10.** Substitutions in the indicated positions of OsGF14e and Nl14 abolish their interaction with OsEDR1l in yeast and *N. benthamiana* cells. A) Full-length amino acid sequences alignment of OsGF14e and TFT7 from tomato. Identical amino acids were shaded gray. The positions of Ala substitutions are underlined in red. B) Y2H assay of yeast strain co-transformed with the indicated plasmids and spotted on quadruple dropout media SD/-Trp/-Leu/-His/-Ade with X-a-gal. C) BiFC analysis to determine the interaction in the mesophyll cells of *N. benthamiana*. Ala substitutions were made at R137 and Y138 positions of OsGF14e to generate OsGF14e (R137A/Y138A). Ala substitutions were made at R130 and Y131 positions of Nl14 to generate Nl14 (R130A/Y131A).


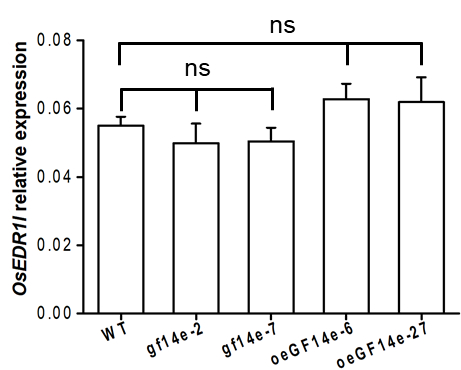


**Figure S11.** OsEDR1l expression in *gf14e* mutants, oeGF14e and WT plants. Mean transcript levels ± SEM (*n* = 3) of *OsEDR1l* expression in *gf14e* mutants, oeGF14e and WT plants. ns, not significant.


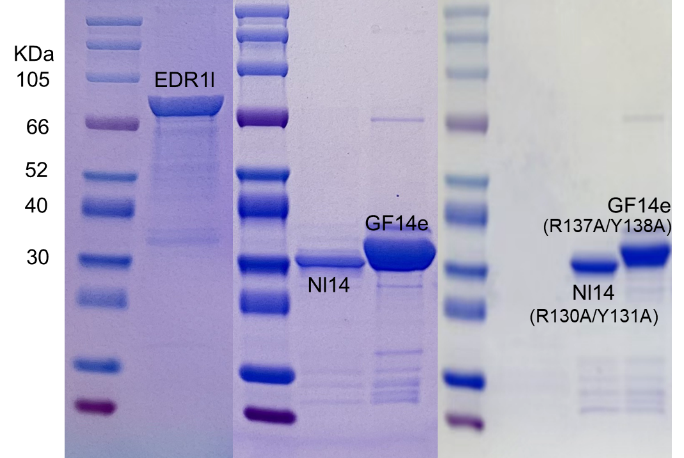


**Figure S12.** The SDS-PAGE analysis of the recombinant EDR1l, Nl14, GF14e, Nl14 (R130A/Y131A) and GF14e (R137A/Y138A) proteins.


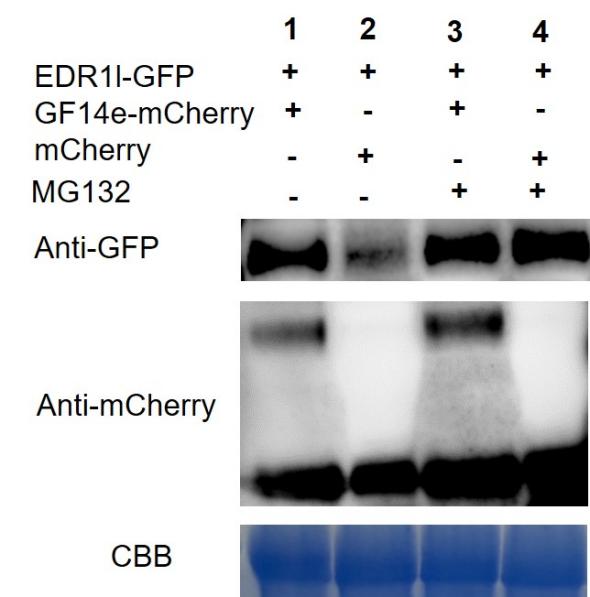


**Figure S13.** The effect of a proteasome inhibitor on OsEDR1l protein abundance in *N. benthamiana* leaves. Lines 1 and 2, western blot assays of EDR1l-GFP fusion protein accumulation in *N. benthamiana* leaves co-expressing OsEDR1l-GFP and OsGF14e-mCherry (or OsEDR1l-GFP and mCherry) indicated that OsGF14e increased accumulation of OsEDR1l. Lines 3 and 4, after 12h, a proteasome inhibitor MG132 was injected at the same infiltrated area. Such difference between line 1 with 2 disappeared in lines 3 and 4. Total proteins were detected using anti-GFP or anti-mCherry. CBB staining was used to estimate protein loading.


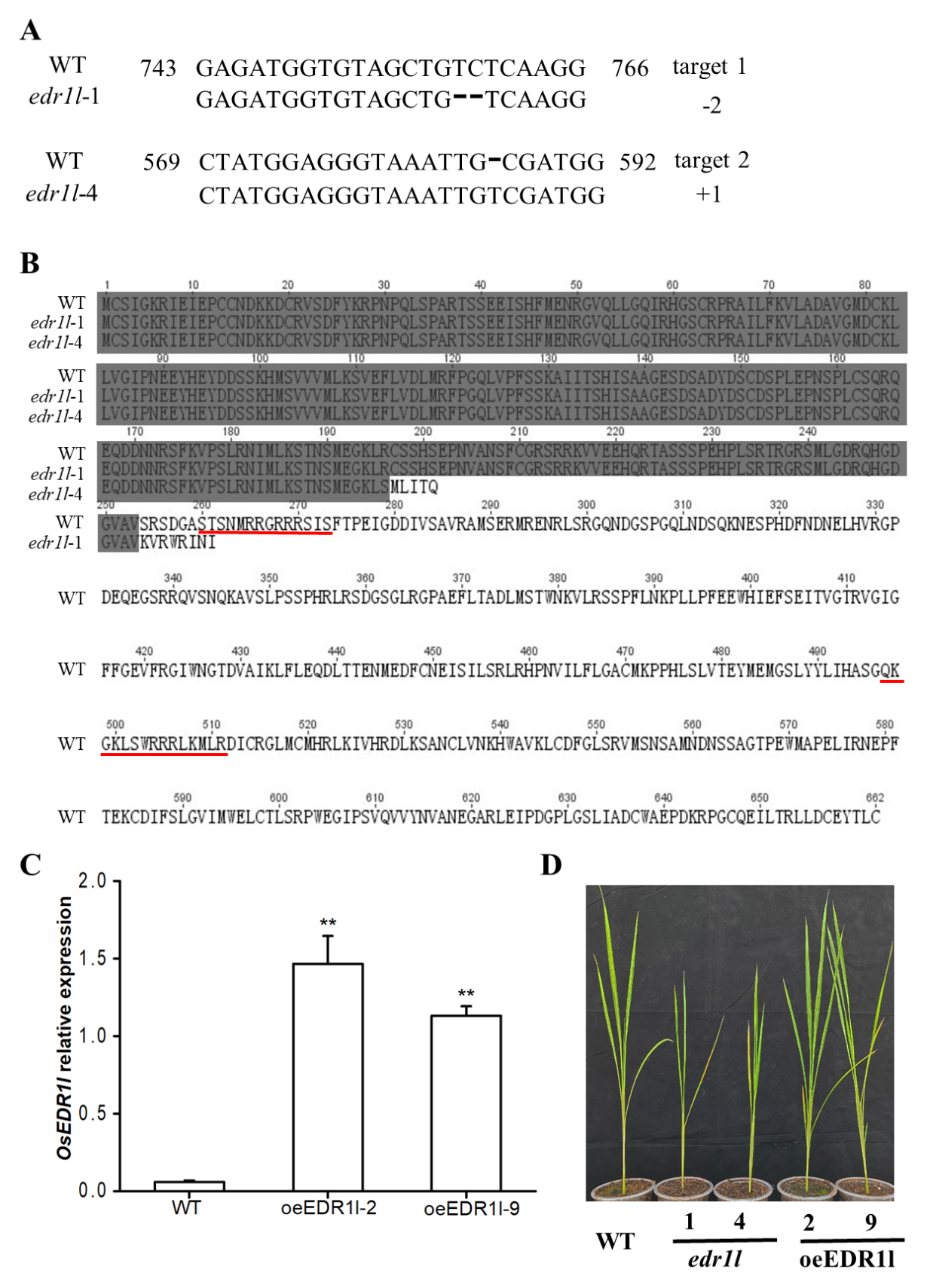


**Figure S14.** Mutation and overexpression of *OsEDR1l* gene. A) Mutation of *OsEDR1l* gene by CRISPR-Cas9. The two target sequences of single-guide RNA (sgRNA) are shown. B) The amino acid sequence of OsEDR1l in *edr1l* mutants and WT plants. The nuclear localization sequences are underlined in red. C) Mean transcript levels ± SEM (*n* = 3) of *OsEDR1l* in oeEDR1l and WT plants. Asterisks indicate significant differences between oeEDR1l and WT plants (**, *p* < 0.01; Student’s *t*-test). D) Phenotypic differences of *edr1l* mutants, oeEDR11 and WT plants.


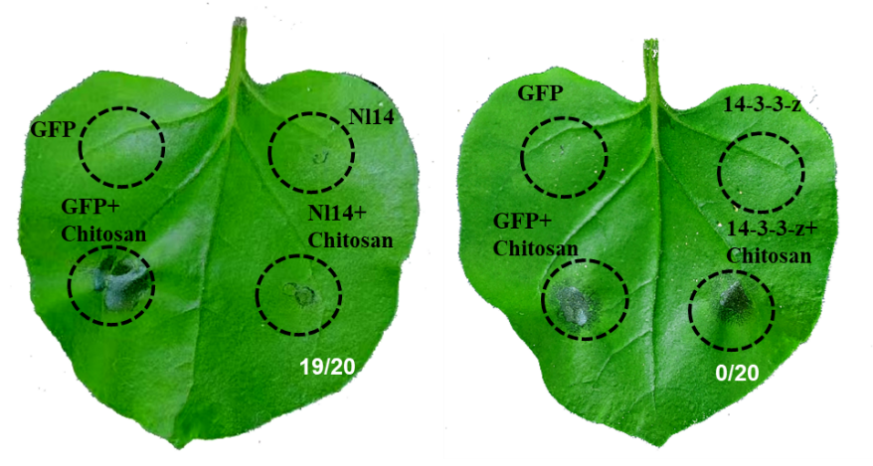


**Figure S15.** Inhibition of chitosan-triggered cell death in *N. benthamiana* leaves by Nl14 expression. Chitosan-induced *N. benthamiana* cell death was observed in all leaf areas expressing GFP. Compared to the control leaf areas, a ratio in the circle of the representative leaf expressing Nl14 or 14-3-3z represented the number of leaf areas showing cell death mitigation relative to the total 20 experimental leaves. Each experiment was repeated with 20 leaves.


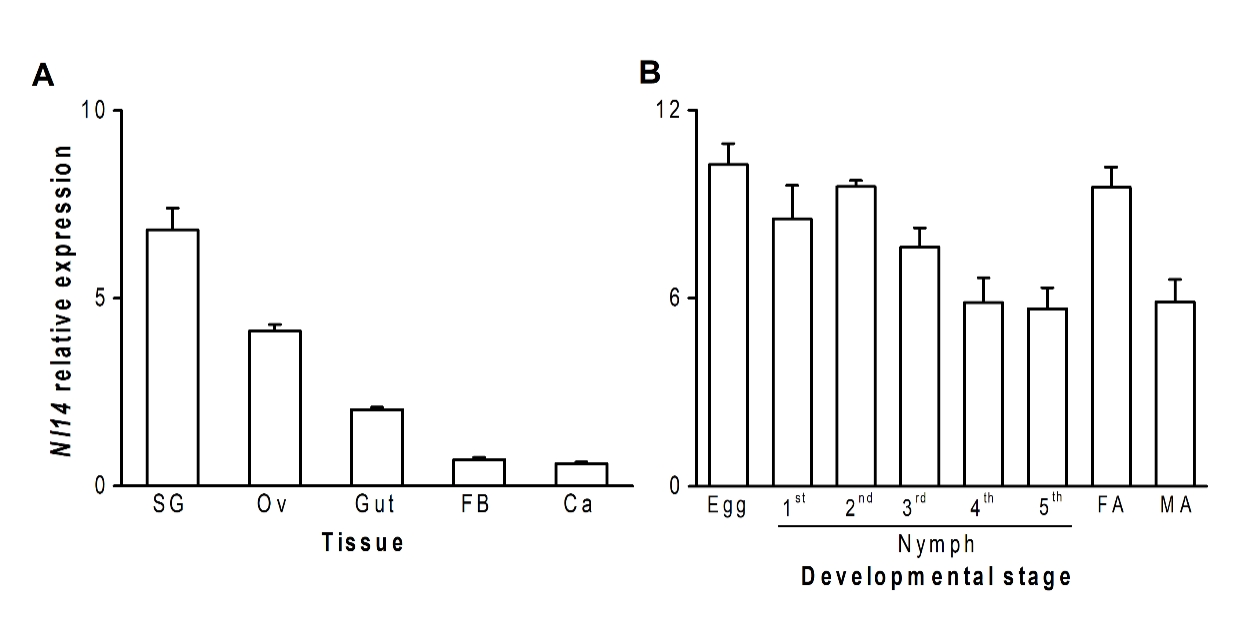


**Figure S16.** Spatiotemporal expression of *Nl14* in BPH. Mean transcript levels ± SEM (*n* = 3) of *Nl14* in BPH gravid female adults in different tissues (A) and at various developmental stages (B). SG, salivary gland; Ov, oviduct; FB, fat body, Ca, carcass. FA, female adults; MA, male adults.


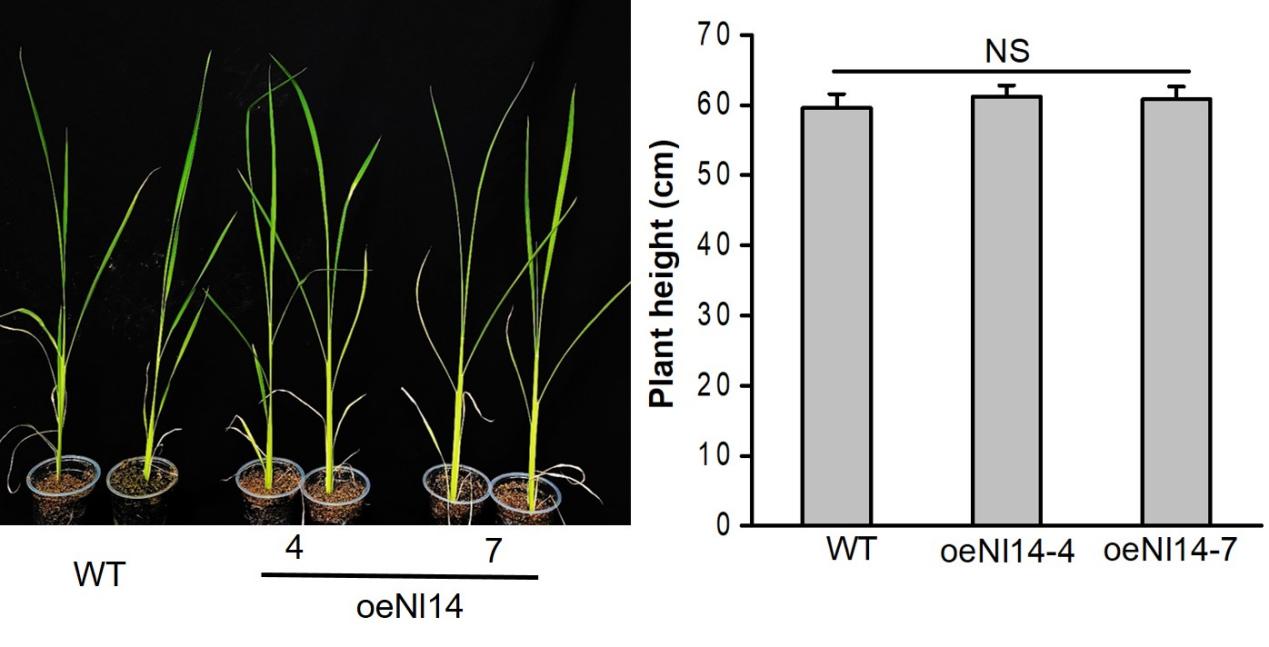


**Figure S17.** Growth phenotypes of oeNl14 and WT plants. Mean plant height ± SEM (*n* = 10) of oeNl14 and WT plants. NS, not significant (Student’s *t*-test).


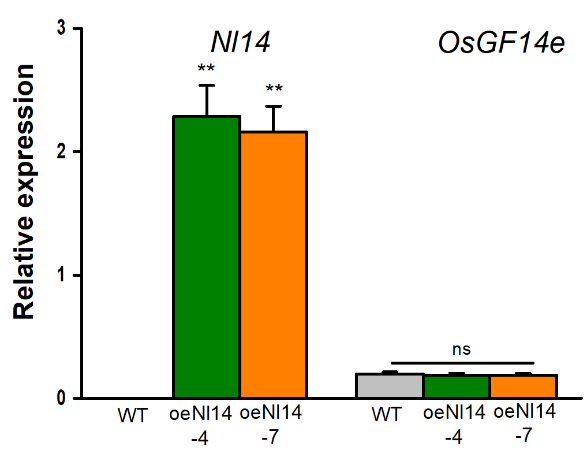


**Figure S18.** *Nl14* and *OsGF14e* expression in oeNl14 and WT plants. Mean transcript levels ± SEM (*n* = 3) of *Nl14* and *OsGF14e* expression in oeNl14 and WT plants. Asterisks indicate significant differences between oeNl14 and WT plants (**, *p* < 0.01; ns, not significant; Student’s *t*-test).


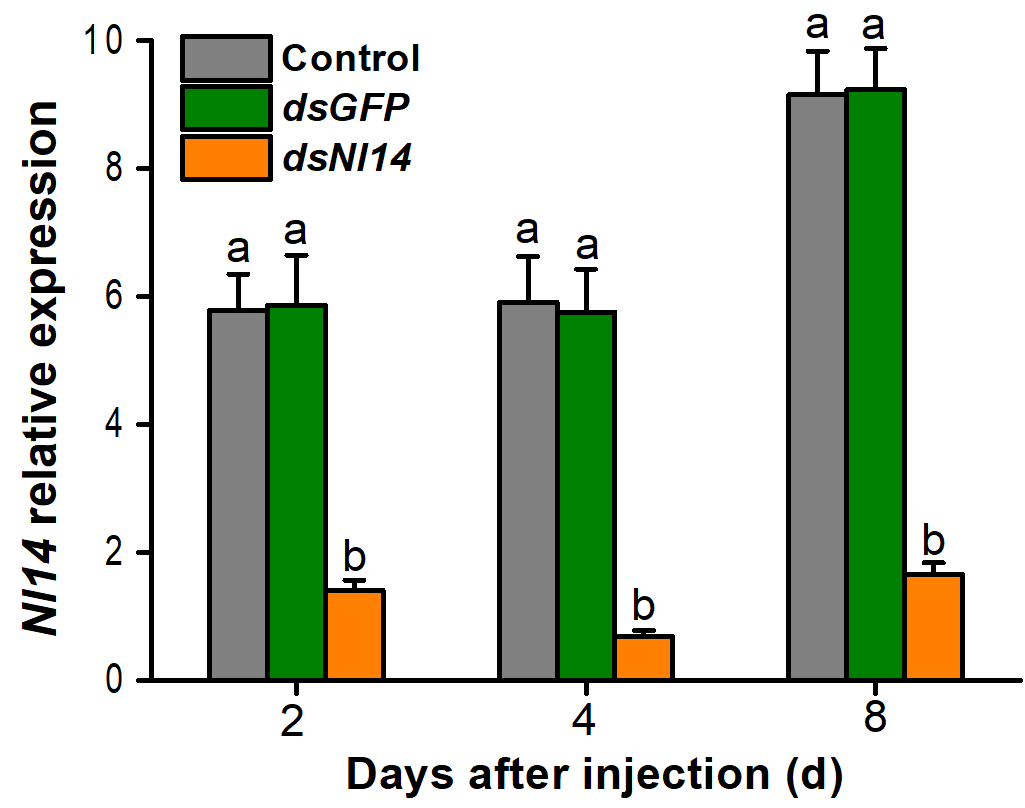


**Figure S19.** *Nl14* silencing by dsRNA-mediated RNAi. Mean transcript levels ± SEM (*n* = 3) of *Nl14* in BPH 2-8 d post-injection. Nymphs were injected with *Nl14* dsRNA (*dsNl14*) or *GFP* dsRNA (*dsGFP*). Controls were untreated insects. The experiment was repeated three times with 6 samples per replicate and 20 insects combined as a sample. Different letters indicate significant difference among treatments (*p*< 0.05, Duncan’s multiple range test).


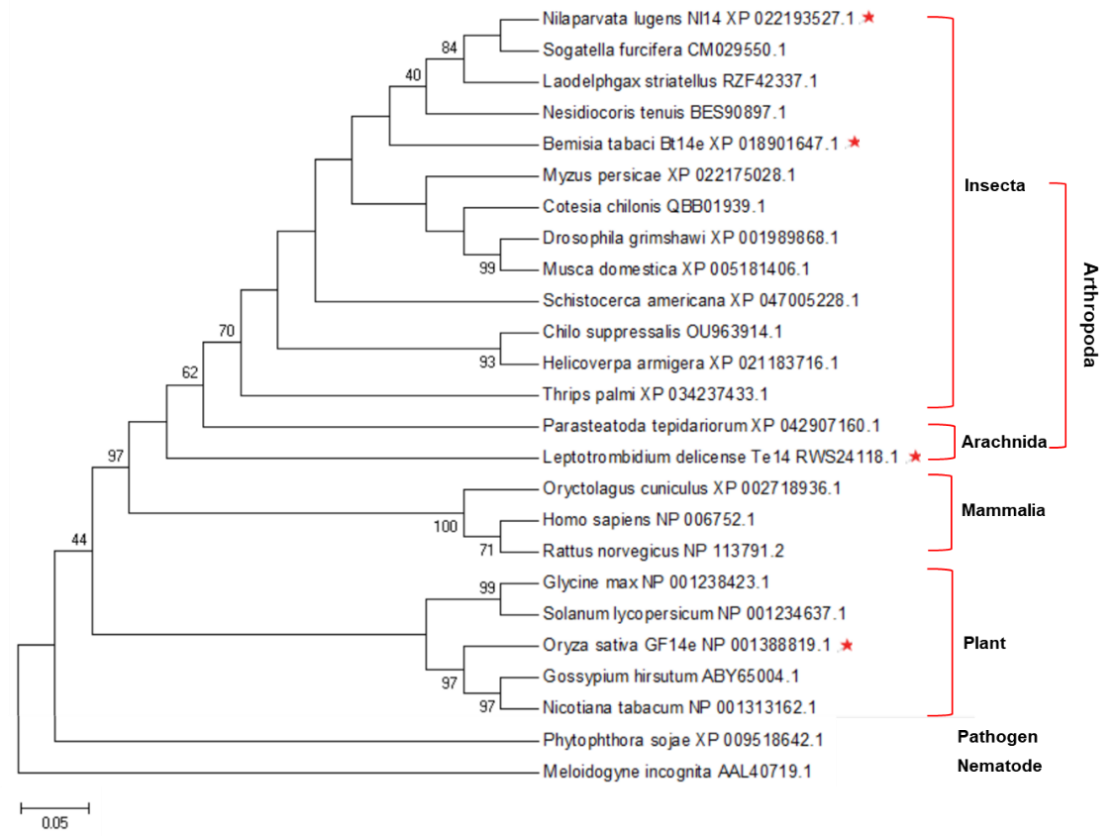


**Figure S20.** Phylogenetic relationships of 14-3-3e members from Arthropoda (insecta and Arachnida), Mammalia, plant, pathogen and nematode. The phylogenetic tree was constructed using MEGA 11.0 with Neighbor-Joining method. The scale bar represents 0.05 amino acid substitutions per site. Bootstrap values at phylogenetically are shown. Nl14, Bt14, Te14 and GF14e are indicated by red stars.


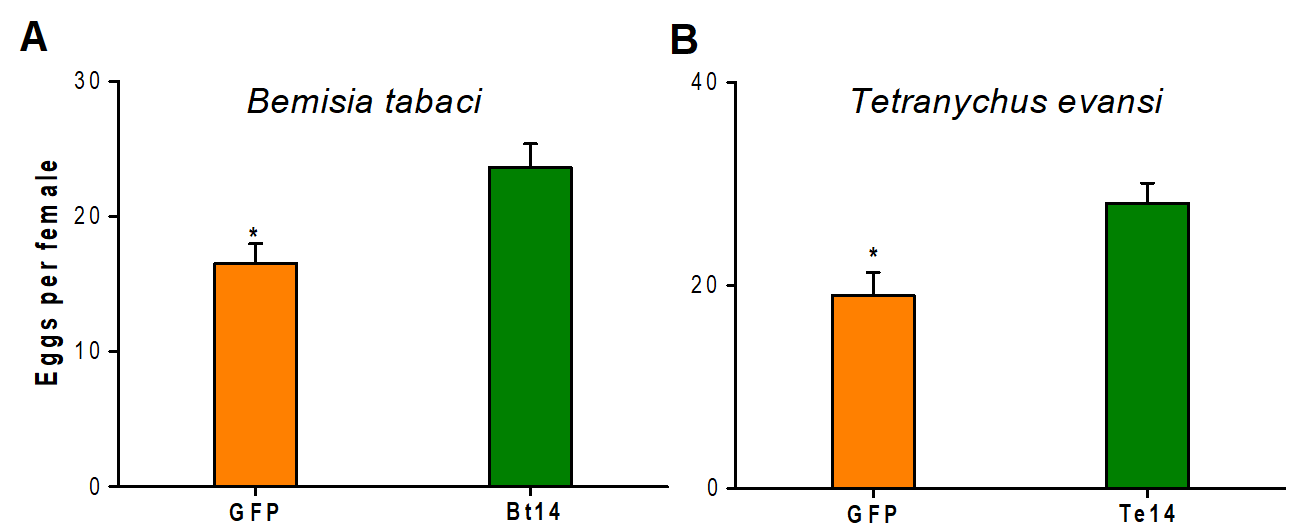


**Figure S21.** Expression of salivary 14-3-3e from *Bemisia tabaci* or *Tetranychus evansi* in *N. benthamiana* promotes insect fecundity. Mean number ± SEM (*n* = 10) of eggs laid by a *B. tabaci* (A) or *T. evansi* (B) female adult fed on *N. benthamiana* leaves expressing GFP or Bt14 or Te14. Asterisks indicate significant difference (*, *p* < 0.05; Student’s *t*-test).

**Figure S22.** Original images of western blots


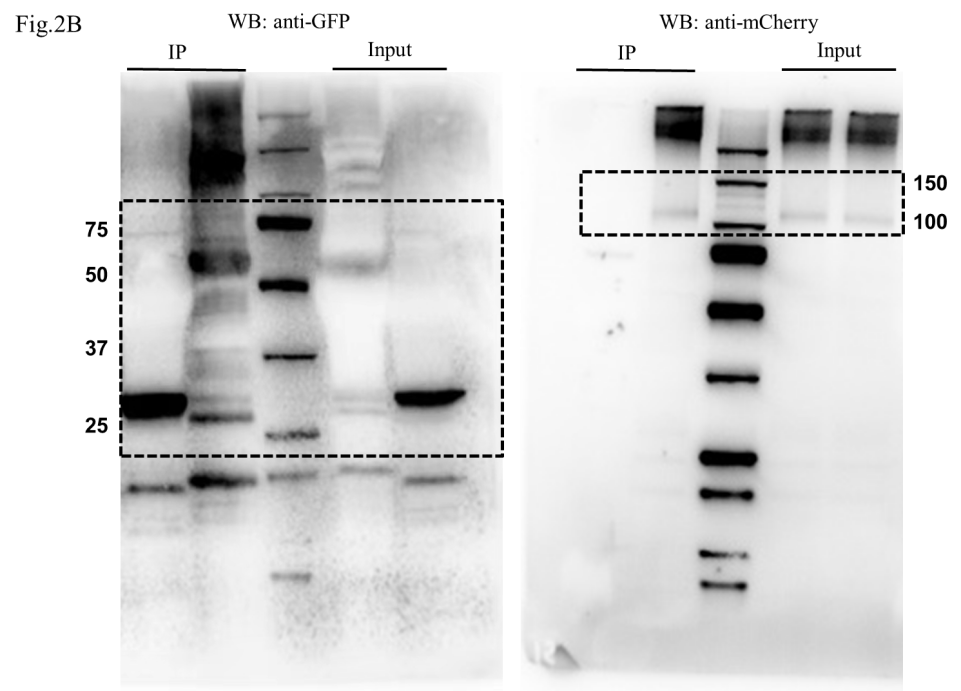


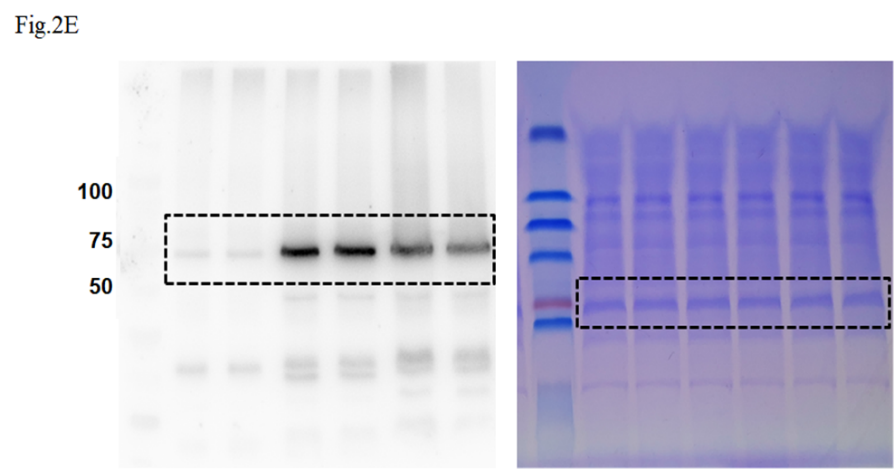


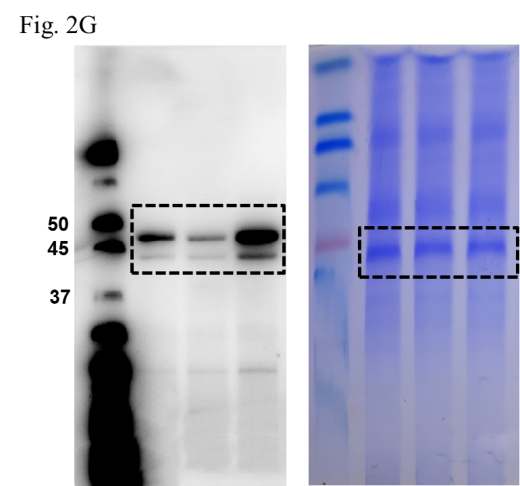


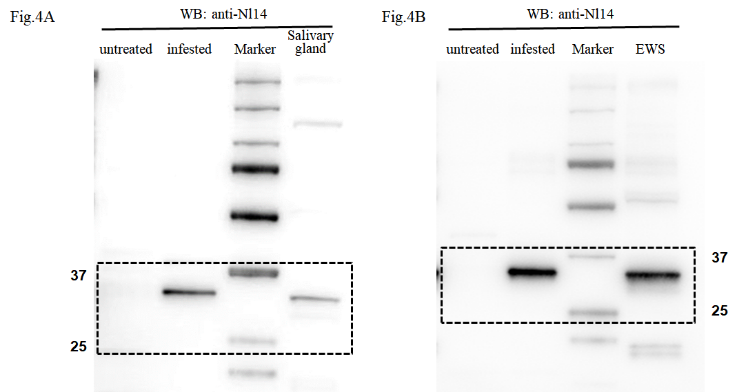


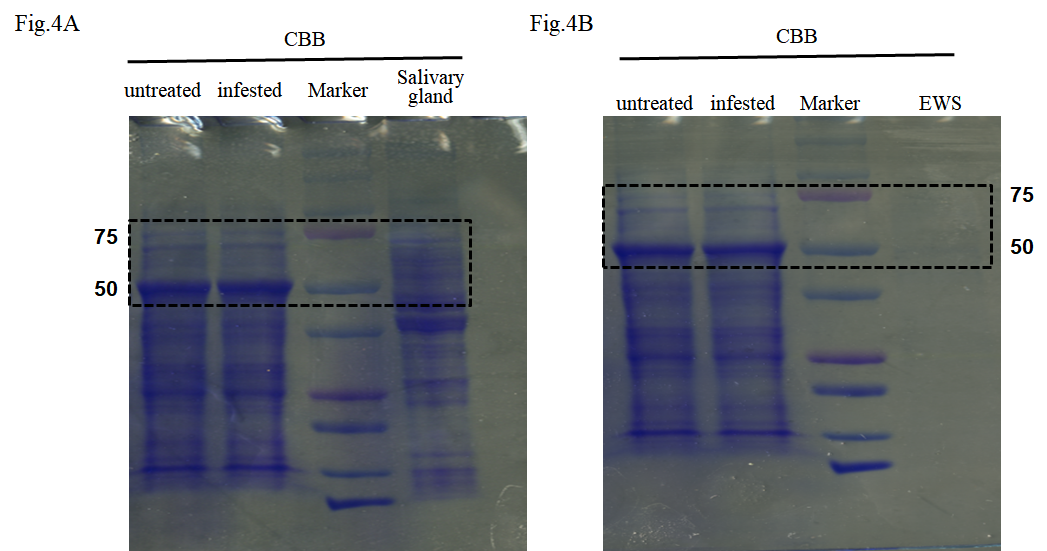


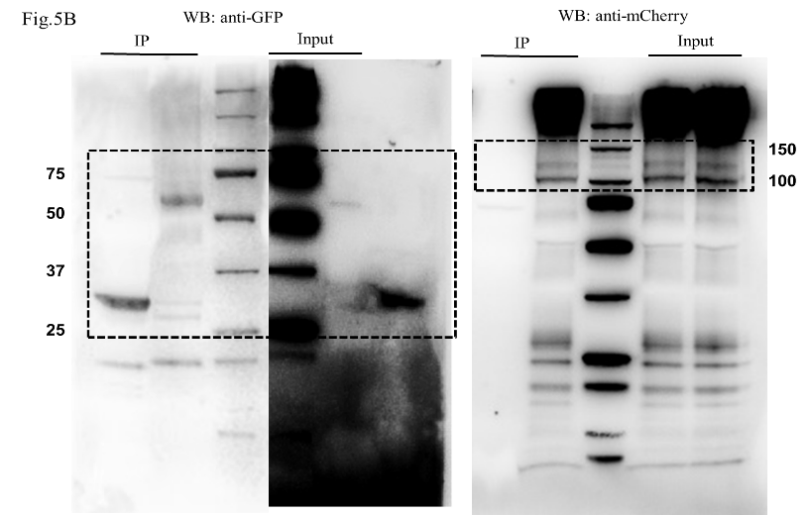


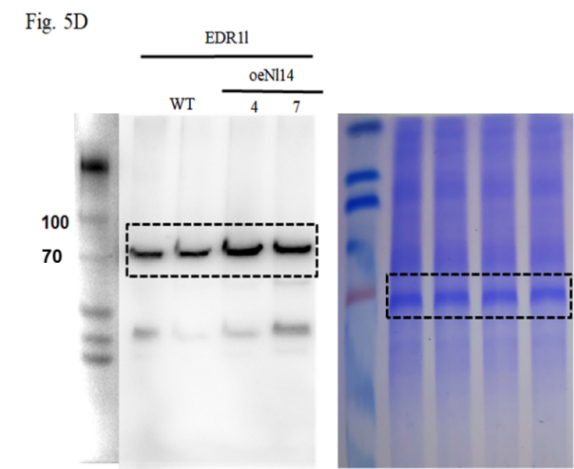


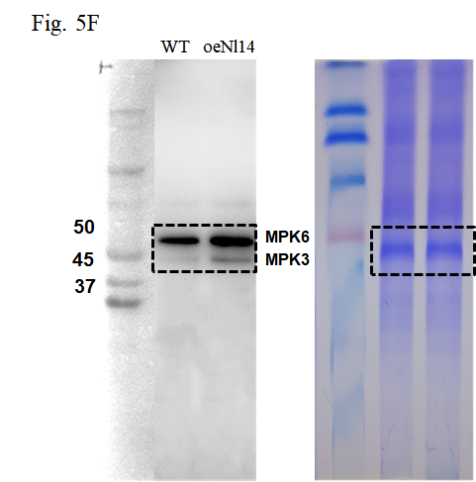
 **Table S1** Rice proteins identified by Y2H screening using OsGF14e as the bait

| **Annotation** | **Colonies** | **GenBank accession** |
| --- | --- | --- |
| 50S ribosomal protein L2, chloroplastic | 1 | XM_026024874.1 |
| 40S ribosomal protein S20-1； | 1 | XM_015759332.2 |
| putative disease resistance protein At3g14460 | 3 | XM_015757266.2 |
| vegetative cell wall protein gp1 | 3 | XM_015782689.2 |
| transport inhibitor response 1-like protein | 2 | XM_015770761.2 |
| FT-interacting protein 1 | 1 | XM_015780862.2 |
| dnaJ protein ERDJ3A-like | 1 | XM_015777241.2 |
| monosaccharide-sensing protein | 1 | XM_015770477.2 |
| LLS1 protein | 1 | AF284781.2 |
| lectin-like protein | 2 | AF435970.1 |
| **serine/threonine-protein kinase EDR1-like** | **5** | **XM_015770804.2** |
| NAD(P)H dehydrogenase (quinone) | 1 | XM_015766024.2 |
| xylose isomerase | 1 | XM_015791332.1 |
| 26S proteasome non-ATPase regulatory subunit 8 | 1 | XM_015792556.2 |
| 14-3-3e | 2 | XM_015770740.2 |
| Unknown protein | 1 | CP018158.1 |
| Unknow protein | 1 | AP014957.1 |
| Unknown protein | 1 | XM_015788595.2 |
| Unknown protein | 2 | XM_015783846.2 |

**Table S2.** Primers used in this study

| **Primers** | **Sequences (5’-3’)** |
| --- | --- |
| **Transient expression/** **Co-IP** |  |
| Nl14-GFP-F | CGGGGTCGACGGATCCATGTCTGAAAGAGAAGACAATGTTT |
| Nl14-GFP-R | TGCTCACCATGGATCCCGACACGTCTTGATCTTCCAC |
| OsGF14e-GFP-F | CGGGGTCGACGGATCCATGTCGCAGCCTGCTGAGC |
| OsGF14e-GFP-R | TGCTCACCATGGATCCCTGTCCATCTCCTGATTCG |
| OsEDR1l-mCherry-F | CGGGGTCGACGGATCCATGTGCAGCATTGGTAAAAGG |
| OsEDR1l-mCherry-R | TGCTCACCATGGATCCACATAAGGTGTATTCGCAGTC |
| **BiFC** |  |
| Nl14-cYFP-F | GCTCGCCTGGGGATCCATGTCTGAAAGAGAAGACAATGTTT |
| Nl14-cYFP-R | CGGGAGATGCGGATCCTTACGACACGTCTTGATCTTCCAC |
| OsGF14e-cYFP-F | GCTCGCCTGGGGATCCATGTCGCAGCCTGCTGAGC |
| OsGF14e-cYFP-R | CGGGAGATGCGGATCCTTACTGTCCATCTCCTGATTCG |
| OsEDR1l-nYFP-F | CGGGAGATGCGGATCCATGTGCAGCATTGGTAAAAGG |
| OsEDR1l-nYFP-R | GCTCGCCTGGGGATCCTTAACATAAGGTGTATTCGCAGTC |
| Os02g12810.1- nYFP-F | CGGGAGATGCGGATCCATGTCACGCATGAAGCATCTG |
| Os02g12810.1- nYFP-R | GCTCGCCTGGGGATCCTCAGCAGTTTTCTGGAACAACTAAC |
| Os03g06410 - nYFP-F | CGGGAGATGCGGATCCATGAAGAATCTGTTCAAGAGTAAGA |
| Os03g06410 - nYFP-R | GCTCGCCTGGGGATCCTCAAGGGGTGGAAGAATTCAC |
| Os06g12590.1- nYFP-F | CGGGAGATGCGGATCCATGAAGAACTTCCTCCGGAAGC |
| Os06g12590.1- nYFP-R | GCTCGCCTGGGGATCCTTAGCCATCTGCTCGTTGCAC |
| Os02g50970.1- nYFP-F | CGGGAGATGCGGATCCATGAAGAACTTCTTCAGGAAGCTCC |
| Os02g50970.1- nYFP-R | GCTCGCCTGGGGATCCTTACTCGTCGGTTTGTTGTACTCTC |
| Os04g52140.1- nYFP-F | CGGGAGATGCGGATCCATGCCTCACCGACGACGC |
| Os04g52140.1- nYFP-R | GCTCGCCTGGGGATCCTCATGAGCCACCAAGCATCG |
| Os10g29540.1- nYFP-F | CGGGAGATGCGGATCCATGAAGATCCCGTTCGTGACC |
| Os10g29540.1- nYFP-R | GCTCGCCTGGGGATCCTCAAACGTGAAAGTCCAAAATCAC |
| Os09g39320.1- nYFP-F | CGGGAGATGCGGATCCATGGACCTCCCCGCCGT |
| Os09g39320.1- nYFP-R | GCTCGCCTGGGGATCCTCATCCAAAAAACATGTCCAAGTC |
| Os02g32610.3- nYFP-F | CGGGAGATGCGGATCCATGAAGGCCGACGCCAAG |
| Os02g32610.3- nYFP-R | GCTCGCCTGGGGATCCTTAACTGATATCCTCTTGAAGTTGA |
| **Y2H** |  |
| Nl14-BD-F | CATGGAGGCCGAATTCATGTCTGAAAGAGAAGACAATGTTT |
| Nl14-BD-R | GGATCCCCGGGAATTCTTACGACACGTCTTGATCTTCCAC |
| OsGF14e-BD-F | CATGGAGGCCGAATTCATGTCGCAGCCTGCTGAGC |
| OsGF14e-BD-R | GGATCCCCGGGAATTCTTACTGTCCATCTCCTGATTCG |
| OsEDR1-AD-F | GGAGGCCAGTGAATTCATGTGCAGCATTGGTAAAAGG |
| OsEDR1-AD-R | CACCCGGGTGGAATTCTTAACATAAGGTGTATTCGCAGTC |
| Os02g12810.1-AD-F | GGAGGCCAGTGAATTCATGTCACGCATGAAGCATCTG |
| Os02g12810.1-AD-R | CACCCGGGTGGAATTCTCAGCAGTTTTCTGGAACAACTAAC |
| Os03g06410-AD-F | GGAGGCCAGTGAATTCATGAAGAATCTGTTCAAGAGTAAGA |
| Os03g06410 –AD-R | CACCCGGGTGGAATTCTCAAGGGGTGGAAGAATTCAC |
| Os06g12590.1-AD-F | GGAGGCCAGTGAATTCATGAAGAACTTCCTCCGGAAGC |
| Os06g12590.1-AD-R | CACCCGGGTGGAATTCTTAGCCATCTGCTCGTTGCAC |
| Os02g50970.1-AD-F | GGAGGCCAGTGAATTCATGAAGAACTTCTTCAGGAAGCTCC |
| Os02g50970.1-AD-R | CACCCGGGTGGAATTCTTACTCGTCGGTTTGTTGTACTCTC |
| Os04g52140.1-AD-F | GGAGGCCAGTGAATTCATGCCTCACCGACGACGC |
| Os04g52140.1-AD-R | CACCCGGGTGGAATTCTCATGAGCCACCAAGCATCG |
| Os10g29540.1-AD-F | GGAGGCCAGTGAATTCATGAAGATCCCGTTCGTGACC |
| Os10g29540.1-AD-R | CACCCGGGTGGAATTCTCAAACGTGAAAGTCCAAAATCAC |
| Os09g39320.1-AD-F | GGAGGCCAGTGAATTCATGGACCTCCCCGCCGT |
| Os09g39320.1-AD-R | CACCCGGGTGGAATTCTCATCCAAAAAACATGTCCAAGTC |
| Os02g32610.3-AD-F | GGAGGCCAGTGAATTCATGAAGGCCGACGCCAAG |
| Os02g32610.3-AD-R | CACCCGGGTGGAATTCTTAACTGATATCCTCTTGAAGTTGA |
| **RNAi in BPH** |  |
| dsNl14-F | TAATACGACTCACTATAGGTTTCCCATCTTGTGCGAG |
| dsNl14-R | TAATACGACTCACTATAGGCGTCAACTCCAGGTCAAG |
| dsGFP-F | TAATACGACTCACTATAGGATGGTAGATCTGACTAGTAA |
| dsGFP-R | TAATACGACTCACTATAGGCTAGTCATCTGCACCTTCTG |
| **RT-qPCR** |  |
| Nl14-qF | AAACTCACTCGGTGCTTGTC |
| Nl14-qR | AGCCTTACTCGACTCTGAATACTG |
| ef2-qF (BPH) | GTCTCCACGGATGGGCTTT |
| ef2-qR (BPH) | ATCTTGAATTTCTCGGCATACATTT |
| OsEDR1l-qF | CAACGCCAAGAGCAAGATGACAAC |
| OsEDR1l-qR | GTGTGATGAGCATCGCAATTTACCC |
| Osactin-qF (rice) | CTGGTATTGCTGACCGTAT |
| Osactin-qR (rice) | GTTGGAAGGTGCTAAGGGA |
| OsGF14a-qF | TCCTACAAGGACAGCACCCT |
| OsGF14a-qR | TCATCCTCAGGCTTGGTTGC |
| OsGF14b-qF | GGCTGTGATTGTTGTCGGGAAA |
| OsGF14b-qR | GAAGTGCCACCAAGCAGTTCAA |
| OsGF14c-qF | TAGATGCCTCATGCTGCTGTCA |
| OsGF14c-qR | AGACGGACCACTAGCACCAAAT |
| OsGF14d-qF | TCAGGACATTGCTCTCGCAGAT |
| OsGF14d-qR | ACCAAGGCTGTCCAGTTCTGAT |
| OsGF14e-qF | AGGATATTGCCCTGGCAGAGTT |
| OsGF14e-qR | TGCAAGATTGCAAGCACGGT |
| OsGF14f-qF | GCATACAAGTCTGCCCAGGA |
| OsGF14f-qR | TCAGTGCAAGTCCAAGCCTT |
| OsGF14g-qF | AGTTGAGCTGCTGAGTAACCCT |
| OsGF14g-qR | AAGAGCGATCAGCGAGTGCTTA |
| OsGF14h-qF | GTGAGCTTTCTCCTGCCCAT |
| OsGF14h-qR | TGCTGTCCTTGTAACCCTCC |
| **Production and purification of recombinant proteins** |  |
| OsEDR1l-GST-F | TTCCAGGGGCCCCTGGGATCCATGTGCAGCATTGGTAAAAGGA |
| OsEDR1l-GST-R | GGCCGCTCGAGTCGACCCGGGTCAACATAAGGTGTATTCGCAGTC |
| OsGF14e-HIS-F | TAAGAAGGAGATATACATATGATGTCGCAGCCTGCTGAGC |
| OsGF14e-HIS-R | CTCGAGTGCGGCCGCAAGCTTTTAATGATGATGATGATGGTGCGAATCAGGAGATGGACAGTGA |
| Nl14-HIS-F | TAAGAAGGAGATATACATATGATGTCTGAAAGAGAAGACAATGTTT |
| Nl14-HIS-R | CTCGAGTGCGGCCGCAAGCTTTTAATGATGATGATGATGGTGCGACACGTCTTGATCTTCCAC |

Homologous recombination sites are underlined.
